# CK2 and protein kinases of the CK1 superfamily as targets for neurodegenerative disorders

**DOI:** 10.3389/fmolb.2022.916063

**Published:** 2022-10-06

**Authors:** Andrea Baier, Ryszard Szyszka

**Affiliations:** Institute of Biological Sciences, The John Paul II Catholic University of Lublin, Lublin, Poland

**Keywords:** neurodegenerative diseases, CK1, CK2, phosphorylation, TTBK, inhibitors

## Abstract

Casein kinases are involved in a variety of signaling pathways, and also in inflammation, cancer, and neurological diseases. Therefore, they are regarded as potential therapeutic targets for drug design. Recent studies have highlighted the importance of the casein kinase 1 superfamily as well as protein kinase CK2 in the development of several neurodegenerative pathologies, such as Alzheimer’s disease, Parkinson’s disease, Huntington’s disease, and amyotrophic lateral sclerosis. CK1 kinases and their closely related tau tubulin kinases as well as CK2 are found to be overexpressed in the mammalian brain. Numerous substrates have been detected which play crucial roles in neuronal and synaptic network functions and activities. The development of new substances for the treatment of these pathologies is in high demand. The impact of these kinases in the progress of neurodegenerative disorders, their bona fide substrates, and numerous natural and synthetic compounds which are able to inhibit CK1, TTBK, and CK2 are discussed in this review.

## Introduction

Due to the fact that the world population is getting progressively older, the risk of neurodegenerative disorders (NDDs) is increasing. Those disorders occur when neurons lose their structure and function which finally leads to their death. Noteworthy, by 2000 it was estimated that the number of patients suffering from dementia in developed countries reached 13.5 million and will rise up to 36.7 million in 2050 (Zheng and Chen, 2022). Alzheimer’s disease (AD) and Parkinson’s disease (PD) are the most prevalent NDDs worldwide.

NDDs are classified according to their major clinical symptoms, altered proteins, and cellular/subcellular pathology. On the molecular level, typical features include protein misfolding and the formation of protein aggregates. The exact mechanisms for that are still unknown. These aggregates are the result of protein modification, like phosphorylation, sumoylation, and ubiquitination (Kovacs et al., 2010).

Typical characteristics of AD are senile plaques of amyloid-beta (Aβ) peptide precipitated in the space between neurons and the neurofibrillary tangles (NFTs) of fibrillar hyperphosphorylated tau protein (Glenner and Wong, 1984; Grundke-Iqbal et al., 1986). The Aβ peptide is the product of the proteolytic cleavage by β- and γ-secretases of the amyloid precursor protein (APP) (Vassar et al., 1999). In AD patients typical clinical features are the progressive loss of mental abilities, e.g., increasing forgetfulness, changes in personality, and cognitive difficulties. Additionally, as result of an immune response in the brain, nerve cells lose their function and die.

Huntington’s disease (HD) is caused by an autosomal dominant genetic defect. Mutations in the *huntingtin* gene (HTT) encoding huntingtin are responsible for the onset of HD. Typical features of this disease are movement disorders, like chorea and loss of coordination, as well as cognitive decline. Furthermore, common psychiatric symptoms are psychosis, depression, and obsessive-compulsive disorder (Rosenblatt, 2007). In HD patients a degeneration of the striatum and general shrinkage of the brain can be observed (Reiner et al., 1988). Loss of cortical mass is regionally selective and proceeds from posterior to anterior cortical regions during HD progression. Other symptoms are weight loss, cardiac failure, and skeletal-muscle wasting (Arenas et al., 1998; Aziz et al., 2008).

PD is a neurodegenerative disease characterized by progressive loss of neuromelanin containing dopaminergic neurons in the substantia nigra pars compacta. There is evidence for the relation between a small volume of this brain region and the weaker or less controlled motor movements of PD patients resulting in the often observed tremors (Menke et al., 2010). The pathological picture of PD includes motor impairments, like resting tremors, bradykinesia, postural instability, and rigidity. Due to the loss of norepinephrine non-movement related symptoms, like psychiatric problems, low blood pressure, and constipation, are seen. The cause of PD might be a combination of environmental and genetic factors.

Amyotrophic lateral sclerosis (ALS) is a fast progressive NDD affecting lower and upper neurons in the brain stem, spinal cord, and the motor cortex (Robberecht and Philips, 2013). It is the most common motor neuron disease in adults and the third most NDD worldwide (Renton et al., 2013). Typical features of ALS are atrophy and paralysis of skeletal muscles resulting from neuron loss and lack of communication between voluntary muscles of the body and the nervous system. Besides these symptoms, in most cases also cognitive and behavioral dysfunctions are present. ALS patients generally die within 3–5 years after first symptoms (Regal et al., 2006). The great majority of cases are classified as sporadic ALS, whereas only 10% are familial. Abnormal aggregations of transactive response DNA-binding protein 43 (TDP-43) are detected in almost all ALS cases (Neumann et al., 2006; Mackenzie et al., 2010). Mutations in the *TARDBP* gene encoding TDP-43 are associated with ALS (Sreedharan et al., 2008).

Protein kinases are encoded by about 2% of all human genes and are capable of phosphorylating up to 20% of all proteins. The counterplay of protein kinases and phosphatases regulates many processes in living cells through modification of serine, threonine and tyrosine residues (Hunter, 1995, 2012; Cohen, 2002; Schwartz and Murray, 2011; Nishi et al., 2014). As a result of phosphorylation protein activities, their stability, localization and interaction with other proteins are controlled. This posttranslational modification is capable of changing protein functions either by allosteric interaction or binding to regulatory domains (Jin and Pawson, 2012; Ardito et al., 2017). It has an important impact on processes, such as DNA replication, transcription and translation, cell metabolism, apoptosis, as well as stress and immunological response (Hunter, 1995; Cohen, 2002; Tarrant and Cole, 2009; Karve and Cheema, 2011). Proteins mainly undergo phosphorylation in the cytosol or in the nucleus (Duan and Walther, 2015).

Currently, there are no drugs available which cure or prevent NDDs, only acute disorders and symptoms are treated.

Numerous protein kinases have been described to play an important role in NDDs (Benn and Dawson, 2020). Unfortunately, most kinase inhibitors are not able to cross the blood-brain-barrier and are, therefore, only suitable for non central nervous disorders. During last decades, there is an increasing interest in the field to develop brain penetrant kinase inhibitors using the approaches from cancer research.

## CK2 and protein kinases of the CK1 superfamily

Human protein kinases have been divided into 10 groups, 9 of them contain an eukaryotic kinase domain (ePK) and the last group is classified as atypical kinases. The majority of protein kinases phosphorylate serine and threonine residues (Ser/Thr kinases), others phosphorylate tyrosine (Tyr kinases). Few kinases are able to modify all three amino acids (dual-specificity kinases). Eukaryotic protein kinases share similarities in the primary sequences and structural features (Hanks and Hunter, 1995; Taylor et al., 1995; Taylor and Kornev, 2011).

CK1 isoforms together with the closely related vaccinia-related kinases (VRKs) and tau tubulin kinases (TTBKs) are classified in a separated group within the Ser/Thr kinase superfamily, whereas CK2 isoforms constitute a subclass of the CMGC group (Knippschild et al., 2005; Perez et al., 2011; Venerando et al., 2014; Fulcher and Sapkota, 2020). Protein kinases of the CK1 group and CK2 completely differ in their structure. At the beginning, they were named according to the phosphorylated *in vitro* protein substrate, casein. They were purified for the first time from soluble extracts of lactating bovine mammary (Waddy and Mackinlay, 1971). Natively isolated enzymes were purified on DEAE-cellulose and called casein kinase 1 and 2 depending on their elution profile (Hathaway and Traugh, 1979). G-CK or Fam20C shows high similarity to the casein kinase found in lactating mammary glands. It has been found in rat liver and brain and phosphorylates casein in the Golgi bodies (Bingham and Farrel, 1974; Lasa et al., 1997). Fam20C is characterized as a secretory kinase phosphorylating secreted proteins, from milk to bone proteins. This is important in the process of biomineralization of bones and teeth (Tagliabracci et al., 2015).

### The role of CK1 in NDDs

CK1 is an evolutionarily conserved and ubiquitously expressed protein kinase. It belongs to second-messenger-independent and constitutively active kinases. CK1 exists in monomeric form with seven isoforms (α, β, γ1, γ2, γ3, δ, and ε) and their alternative splicing forms, which are encoded by different genes (Fulcher and Sapkota, 2020). The CK1 isoforms differ in their kinase activities, functions, subcellular localization, and biochemical properties (Zhang et al., 1996; Burzio et al., 2002; Takano et al., 2004; Xu et al., 2019). They vary in their molecular weights between 37 and 51 kDa with CK1α being the smallest and CK1γ3 the largest protein. The analysis of the substrate specificity of CK1 isoforms initially determined pS/pT-X_1-2_-**S**/**T** as their consensus sequence. This led to the assumption that phosphorylation by CK1 depends on the prior phosphorylation of position −2 or −3 (Meggio et al., 1991; Meggio et al., 1992). Further studies have shown that this hypothesis did not hold true when proteins were efficiently phosphorylated without prephosphorylated residues (Flotow and Roach, 1991; Marin et al., 1994; Pulgar et al., 1999). Later, novel substrates were described containing a non-canonical motif (S-L-S) with acidic residues downstream of the phosphorylation site. Over 140 substrates are described for CK1. Most of them are involved in various cell processes, e.g., membrane transport and trafficking, microtubule-associated dynamics, apoptosis, and cell cycle progression (Yang et al., 2017; Xu et al., 2019). In diverse studies the important role of CK1 in NDDs was shown, emphasizing on tauopathies, such as AD. A distribution study of all CK1 isoforms comparing AD and control brains revealed that CK1 can be found in fibrillar lesions and, additionally, within the matrix of granulovacuolar degeneration bodies (Ghoshal et al., 1999; Kannanayakal et al., 2006). Contrary to CK1α which is linked to fibrillar lesions, CK1δ is linked to granulovacuolar degeneration bodies (Kannanayakal et al., 2006). It is possible that alteration of CK1δ function is aligned with dysregulation of circadian rhythms in AD. Elevated expression levels of CK1δ (33-fold) and CK1ε (9-fold) has been described in AD post-mortem brain tissue and ALS (Ghoshal et al., 1999; Yasojima et al., 2000; Salado et al., 2014; Palomo et al., 2020; Carter et al., 2021). Many proteins related to neurological disorders are modified by CK1α, e.g., β-secretase (Walter et al., 2001), α-synuclein (Mbefo et al., 2015), and parkin (Yamamoto et al., 2005). Similarly, it has been discovered that CK1δ phosphorylates several proteins that are associated with different NDDs *in vitro.* CK1δ phosphorylates the tau protein leading to its aggregation and finally the formation of neurofibrillary tangles (Li et al., 2004). Increased CK1 activity is associated with tau aggregation (Schwab et al., 2000). CK1δ-dependent phosphorylation has been also shown for other proteins connected with neurodegenerative diseases, e.g., presenilin-2, β-secretase, parkin, TDP43, α-synuclein, LRRK2, and tau (Okochi et al., 2000; Walter, 2000; Rubio de la Torre et al., 2009; Alquezar et al., 2016; Nonaka et al., 2016; Morales-Garcia et al., 2017; De Wit et al., 2018). Therefore, inhibition of CK1δ/ε has been described to possess favorable effects on ALS, frontotemporal dementia (FTD), and PD phenotypes *in vivo* (Perez et al., 2011; Salado et al., 2014; Alquezar et al., 2016; Cozza and Pinna, 2016; Jiang et al., 2018; Palomo et al., 2020).

### The role of TTBK in NDDs

Within the CK1 superfamily a small family of brain-specific kinases phosphorylating microtubule-associated proteins tau and tubulin is classified (Ikezu and Ikezu, 2014). TTBK is a Ser/Thr and Tyr dual-kinase conducting multiple functions inside the cell. It comprises two isoforms: TTBK1 and TTBK2. TTBK1 was characterized as a neuron-specific kinase phosphorylating tau which leads to its aggregation, while TTBK2 was purified from the bovine brain (Takahashi et al., 1995; Sato et al., 2006). Both isoforms are encoded by distinctive genes, and furthermore, their localization in tissues is diverse (Nozal and Martinez, 2019). The sequence of TTBK1, consisting of 1321 amino acids, can be divided into kinase domain (residues 34-297), and a regulatory domain, which contains a characteristic 39 amino acids poly-Glu motif (Ikezu and Ikezu, 2014). The comparison of the TTBK1 and CK1δ kinase domain sequences revealed a 38% identity and 52% similarity (Sato et al., 2006). TTBK1 is primarily expressed in the human brain, notably in the adult brain cortex, cerebellum, and fetal brain. When mouse brain was analysed, TTBK1 was also detected in the frontal cortical layers, the hippocampus, and the granular layer of the cerebellum. Studies using antibodies confirmed the colocalization with tubulin in neurons (Sato et al., 2006). TTBK1 is upregulated in AD cases (Takashima et al., 1993).

TTBK2 consists of 1244 amino acids, and contrary to TTBK1, is ubiquitously expressed in the whole body. Highest *TTBK2* mRNA expression levels are monitored in cerebellum Purkinje cells, granular cell layer, hippocampus, midbrain, and substantia nigra, whereas lower levels were found in the cortex of human, rat, and mouse brains (Houlden et al., 2007). In analyses of the protein expression in the brain and testis higher amounts of TTBK2 were found which correlates with higher activities of TTBK2 in these tissues (Bouskila et al., 2011). Mutations in the *TTBK2* gene are responsible for the onset of spinocerebellar ataxia type 11, an NDD characterized by progressive ataxia and atrophy of the cerebellum and brainstem.

Both isoforms contain highly similar catalytic domains (88% identity and 96% similarity), but diverse C-terminal domains of 43% identity and 58% similarity (Ikezu and Ikezu, 2014; Nozal and Martinez, 2019). TTBK1/2 were described as kinases which show higher phosphorylation activity in case of a prephosphorylated substrate at position -3 (S/T-X-X-**S/T/Y**). On the surface of TTBK1 two positive sequences have been identified, which might act as putative binding sites for the prephosphorylated substrate (Xue et al., 2013; Kosten et al., 2014). Phosphorylation of tau was shown at Y197, S198, S199, S202, and S422, the critical sites in paired helical filaments (Grundke-Iqbal et al., 1986; Sato et al., 2006; Tomizawa et al., 2001). Interestingly, both isoforms differ in the aa sites which they phosphorylate. Due to tau phosphorylation in neurons at S422, TTBK1 is responsible for neurofibrillary pretangle formation and subsequent tau aggregation (Sato et al., 2008; Vazquez-Higuera et al., 2011; Yu et al., 2011; Lund et al., 2013). In knock-down experiments it has been demonstrated that TTBK1, not TTBK2, is the main isoform responsible for tau phosphorylation at S422 (Bao et al., 2021). Overexpression of TTBK1 in mice resulted in increased phosphorylation and oligomerization of tau in the brain (Xu et al., 2010).

Besides the phosphorylation of tau and tubulin by TTBK2, further substrates include centrosomal proteins CEP164 and CEP97, SV2A as well as neurodegeneration-associated protein TDP-43 (Takahashi et al., 1995; Tomizawa et al., 2001; Ikezu and Ikezu, 2014; Liachko et al., 2014; Liao et al., 2015; Zhang et al., 2015). Additionally, TTBK2 is crucial for the regulation of the growth of axonemal microtubules in ciliogenesis (Liao et al., 2015).

Especially TTBK1 is a promising candidate as target for NDDs treatment. It is mainly expressed in brain tissue, and therefore, possesses limited off-pathway roles. The phosphorylation of tau and TDP-43 makes it an ideal kinase in the case of these two proteinopathies.

### The role of CK2 in NDDs

Together with CK1, CK2 was identified as phosphotranferase using casein as protein substrate for enzymes able to catalyse phosphate transfer from ATP to proteins (Burnett and Kennedy, 1954). Native protein kinase CK2 exists as a heterotetrameric holoenzyme consisting of two catalytic subunits, α and α′, and a dimer of regulatory subunits β (Hathaway and Traugh, 1979). The two isoforms of the CK2 catalytic subunit are highly homologous, but they are products of two different genes (Wirkner et al., 1994; Yang-Feng et al., 1994; Ackermann et al., 2005). CK2 subunits may build different active holoenzymes in three different conformations (αα′, α_2_, or α′_2_) or exist as free catalytic subunits. Each CK2 isoform possesses characteristics common for CK2, but differences in their substrate specificity and sensitivity to inhibitors have been described. They may also regulate different cellular processes (Pinna, 2002; Domańska et al., 2005; Janeczko et al., 2012).

CK2 is a constitutively active protein kinase, independent from second messengers, and is able to use both, ATP and GTP, as phosphoryl donors (Litchfield, 2003). Analysis of the eukaryotic phosphoproteome revealed that CK2 is responsible for the phosphorylation of almost one-quarter of phosphoproteins (Meggio and Pinna, 2003; Salvi et al., 2009; Franchin et al., 2015).

The minimal consensus sequence of CK2 was estimated as **S**/**T**-X-X-D/E/pS/pY, which is present in numerous proteins (Meggio et al., 1994; Venerando et al., 2014).

After the detection of the critical role in various disease states, like cancers and neurodegenerative disorders research groups worldwide focussed their attention on CK2 as a potential therapeutic target (Blanquet, 2000; Perez et al., 2011; Castello et al., 2017; Borgo et al., 2021a).

Several CK2 targets in NDDs were described. α-synuclein is phosphorylated at S129 that leads to aggregates which are the main component of Lewy bodies. In 90% of PD samples this phosphorylation is found, whereas in only 4% of normal tissue. As shown, this site is affected by several kinases and dependent on which one the biological effects might differ (Oueslati, 2016).

In different reports the diverse role of CK2 in AD is described. CK2 phosphorylates presenilin-2 at S7 and S9 *in vitro* while not altering APP cleavage by γ-secretase (Walter et al., 1996; Sannerud et al., 2016). Additionally, it was shown that CK2 phosphorylates SET, a phosphatase PP2A inhibitor, at position S9 which leads to its translocation to the cytoplasm (Zhang et al., 2018).

Numerous reports reveal that CK2 possesses a protective role in HD. The phosphorylation of huntingtin at S13 and S16 alters its location. Phospho-huntingtin is found in the nucleus which reduces its cellular toxicity (Atwal et al., 2011). Similarly, TDP-43 phosphorylation may prevent protein aggregation of truncated forms (Li et al., 2011).

## Proteins involved in neurodegenerative diseases

A lot of NDD-associated proteins playing an important role in the onset of these disorders were identified (Kovacs et al., 2010): (1) the tubulin-associated unit (tau) protein; (2) amyloid-β (Aβ), peptides which result from cleavage of a large transmembrane precursor protein (Aβ-precursor protein or APP); (3) α-synuclein; (4) prion protein; (5) TDP-43 (Ou et al., 1995); (6) fused in sarcoma protein, Ewing’s sarcoma RNA-binding protein 1, and TATA-binding protein-associated factor 15, also known as FET proteins (Neumann et al., 2011). Other proteins are associated with neurological disorders caused by mutations leading to trinucleotide repeats (e.g., huntingtin, ataxins, atrophin-1).

Neurodegenerative proteinopathies can be classified according to the major protein involved in the disease: tauopathies, α-synucleinopathies, TDP-43 proteinopathies, FUS/FET proteinopathies, prion diseases, trinucleotide repeat diseases, neuroserpinopathy, ferritinopathy, and cerebral amyloidoses.

Several of these proteins were identified as substrates for CK1, TTBK, and CK2 and are further described below. Table 1 summarizes information about these proteins phosphorylated by CK1, TTBK, and CK2 and their respective phosphorylation sites.

**TABLE 1:** Selected proteins involved in neurodegenerative diseases and their phosphomodifications by casein kinases.

Protein	Disease	Protein kinase	Phosphorylated residue	References
α-synuclein	Parkinson’s disease, Alzheimer’s disease, Lewy Body Dementia	CK1δ	S87, S129	Okochi et al. (2000)
		CK2	S129	Okochi et al. (2000); Fujiwara et al. (2002); Ishii et al. (2007); Paleologou et al. (2008); Waxman and Giasson, (2008); Xu et al. (2015)
Amyloid-beta precursor protein (APP)	Parkinson’s disease, Alzheimer’s disease	CK1δ/ε, CK2	S198, S206	Walter et al. (2000); Sundaram et al. (2019)
β-Secretase	Alzheimer’s disease	CK1δ/ε	S498	Walter et al. (2000)
Tau protein	Alzheimer’s disease, Parkinson dementia syndrome, Pick disease of the brain	CK1δ/ε	T17, S46, T50, T95, T101, T102, S113, S131, T149, T169, S184, S198, S208, S210, S212, S237, S238, S262, T263, S285, S289, S293, S305, S341, S352, S356, S361, S373, S386, S396, S404, S412, S413, T414, S416, S433, S435	Chen G et al. (2017); Oliveira et al. (2017)
		TTBK1/2	Y197, S198, S199, S202, S205, S208, S210, S416, S422, T427	Tomizawa et al. (2001); Sato et al. (2006); Oliveira et al. (2017); Taylor et al. (2018); Ikezu et al. (2020)
		CK2	T39, T52, S56, S199, S386, S396, S400, S404, S412, S413, S414, S416	Hanger et al. (2007); Greenwood et al. (1994); Oliveira et al. (2017)
TDP-43	Amyotrophic lateral sclerosis (ALS), Frontotemporal lobar degeneration	TTBK1/2	S409/410	Hasegawa et al. (2008); Liachko et al. (2014); Taylor et al. (2018); Taylor et al. (2019)
		CK1δ, CK2	S379, S403/404, S419/410	Kametani et al. (2009); Taylor et al. (2019)
Parkin	Parkinson’s disease	CK1δ	S101, S127, S378	Chakraborty et al. (2017); Yamamoto et al. (2005); Rubio Rubio de la Torre et al. (2009)
Huntingtin	Huntington’s disease	CK2	S13, S16	Atwal et al. (2011)
Ataxin-3	Spinocerebellar ataxia 3 (SCA3)	CK2	S340, S352	Mueller et al. (2009)
Presenilin -2	Alzheimer’s disease	CK2	S7, S9	Walter et al. (1996); Sannerud et al. (2016); Borgo et al. (2021b)
		CK1	S19	Walter et al. (1996); Sannerud et al. (2016)
I_2_^PP2A^	Alzheimer’s disease	CK2	S9	Zhang et al. (2018)

### α-synuclein

The name of this protein is derived from synaptic vesicles (syn-) and the nuclear envelope (-nuclein), both places where α-synuclein was first identified (Maroteaux et al., 1988). As we know now, this was probably the effect of a contaminated antibody that was used in this study. It is involved in PD, dementia with Lewy bodies, and multiple system atrophy (Spillantini et al., 1997; Wakabayashi et al., 1997; Gai et al., 1998). α-synuclein is a ubiquitously expressed protein with the highest levels in neurons, especially in presynaptic terminals. Other localizations for α-synuclein have been also identified, mainly based on overexpression experiments, but its function there remains unclear.

α-synuclein is a protein built of 140 amino acids that undergo posttranslational modification, especially at its C-terminus, e.g., phosphorylation, oxidation, ubiquitination, acetylation, and glycosylation. The major phosphorylation sites are S87 and S129 (Okochi et al., 2000; Fujiwara et al., 2002; Ishii et al., 2007). It was also shown that tyrosine residues are phosphorylated: Y125, Y133, and Y136 (Ellis et al., 2001; Nakamura et al., 2001; Negro et al., 2002). In pathological states, α-synuclein adopts a β-sheet conformation, which consequently leads to α-synuclein aggregation, fibril formation, and deposition into Lewy bodies (Conway et al., 1998; El-Agnaf et al., 1998; Narhi et al., 1999; Uversky, 2007; Yonetani et al., 2009). In PD, Lewy bodies, point mutations, and duplications/triplication of *α-synuclein* gene are the main pathological hallmark (Burré et al., 2018). Lewy bodies containing α-synuclein were also deteced in samples from familial AD patients (Lippa et al., 1998). Furthermore, in senil plaques in AD a short fragment of α-synuclein (aa residues 61-95) was found and termed non-Aβ-amyloid component, a region that is necessary for α-synuclein aggregation and fibrillogenesis (Ueda et al., 1993). Phosphorylation prevents or at least decreases the aggregation and toxicity of α-synuclein (Waxman and Giasson, 2008). Within all identified phosphorylation sites *in vivo* and *in vitro* only S87 lies in the non-Aβ-amyloid component (El-Agnaf et al., 1998).

### Amyloid-beta precursor protein

APP was isolated and purified from cerebral Aβ deposits in 1984 (Glenner and Wong, 1984). It is found in different tissues, particularly in the brain, as a type I transmembrane protein located predominantly in the endoplasmic reticulum (Zheng and Koo, 2006). In 1992, Hardy and Higgins presented the amyloid cascade hypothesis as their theory of AD pathophysiology (Hardy and Higgins, 1992). Proteases from the secretase family (β-secretase and γ-secretase) cleave APP into Aβ peptides of different lengths, mainly Aβ38, Aβ40, and Aβ42, whereas α- and γ-secretases produce P3 peptides (LaFerla et al., 2007; O’Brien and Wong, 2011). Although the most abundant form is Aβ40 (80%–90%), Aβ42 is mainly responsible for protein aggregations and the formation of oligomers, amyloid fibrils, and amyloid plaques (Chen C et al., 2017). Those amyloid plaques are the cause of neurotoxicity in AD progression (Citron et al., 1996; Murphy and LeVine, 2010). Effects of Aβ40/Aβ42 aggregation, especially Aβ oligomers, are calcium dishomeostasis, disturbance of ion channels, alteration of glucose regulation and oxidative damage (Tiwari et al., 2019). Furthermore, it was described that Aβ aggregation promotes tau phosphorylation and aggregation. Out of 30 mutations described in the *APP* gene, 25 are involved in the deposition of insoluble Aβ, like KM670/671NL (Swedish), V717I (London), V717F (Indiana).

### Tau protein

The tau protein was firstly discovered in porcine brain and isolated as heat-stable protein. The function of tau is the stabilization of internal microtubules (Weingarten et al., 1975). It is particularly highly expressed in axons of neuronal cells of the central nervous system (Binder et al., 1985). Studies have shown that tau is a phosphoprotein that then negatively influences the microtubule assembly by changes of the molecule shape (Jameson et al., 1980; Lindwall and Cole, 1984). Phosphorylation of tau is often accompanied by other posttranslational modifications, e.g., *O*-glycosylation, ubiquitination, and methylation. Tau inclusions occur in AD, Pick’s disease, progressive supranuclear palsy, corticobasal degeneration, argyrophilic grain disease, Parkinsonism-dementia complex of Guam, and FTD (Lee et al., 2001).

Tau primary transcript generates six isoforms by alternative splicing resulting in proteins of 352-441 amino acids and MW of 45–65 kDa (Boyarko and Hook, 2021). Tau protein possesses 80 S/T and 5 Y residues of which at least 46 have been found to be phosphorylated in AD (Hanger et al., 2009). The total phosphorylation level of tau in AD and other tauopathies is several times higher than in control samples (Gong and Iqbal, 2008). The ability of tau to polymerize tubulin and to promote microtubule assembly is reduced through hyperphosphorylation (Yoshida and Ihara, 1993). A correlation between the level of hyperphosphorylation at multiple sites and the severity of NFT pathology was found which also correlates with the degree of neuronal loss and cognitive deficit (Grundke-Iqbal et al., 1986; Braak and Braak, 1991; Augustinack et al., 2002).

### TDP-43

TDP-43 is a ubiquitous protein belonging to the ribonucleoprotein family and is normally localized in the nucleus where it takes part in RNA regulation (Nakielny and Dreyfuss, 1997; Geuens et al., 2016). Firstly, it was identified as a transcrptional repressor of HIV-1 transactivator response (TAR) long terminal repeats (Ou et al., 1995). Later, it was demonstrated that it is the major component of ubiquitinated inclusions in ALS and frontotemporal lobar degeneration (FTLD). Posttranslational modifications, such as cleavage, hyperphosphorylation, and ubiquitination lead to cytoplasmic accumulation and aggregation of TDP-43 (Arai et al., 2006; Neumann et al., 2006; Hasegawa et al., 2008). The sequence of TDP-43 is divided into three parts: an N-terminal domain (residues 1–103), two RNA recognition motifs (residues 104–200 and 191–262), and a C-terminal domain (residues 274–413). TDP-43 possesses 64 potential phosphorylation sites. Phosphorylation at S403/404 and S409/410 at the C-terminus results in pathological inclusions (Hasegawa et al., 2008; Inukai et al., 2008; Zhang et al., 2009). The N-terminus contains a nuclear localization sequence that is prone to mutations leading to cytoplasmic localization of TDP-43 and aggregation, whereas the C-terminus is necessary for solubility and cellular localization (Ayala et al., 2008; Barmada et al., 2010).

### Parkin

Parkin possesses an activity of an E3 ubiquitin ligase (Shimura et al., 2000). Insoluble parkin, resulting from point mutations, plays a major role in the inactivation of the protein in PD (Cookson et al., 2003; Sriram et al., 2005; Hampe et al., 2006). Phosphorylation of S101, S127, and S378 was identified using CK1 *in vitro* and *in vivo* with HEK293T cells transiently transfected with parkin (Yamamoto et al., 2005; Rubio de la Torre et al., 2009). Treatment of those cells with IC261, a selective CK1 inhibitor, significantly decreased the phosphorylation level of parkin (Mashhoon et al., 2000; Bain et al., 2007; Rubio de la Torre et al., 2009). Another potent CK1 inhibitor, D4476, was used, which confirmed the hypothesis that S101 and S378 are phosphorylated *in vivo*. Besides CK1, parkin is phosphorylated by CDK5 at S121 (Avraham et al., 2007). Experiments *in vivo* and *in vitro* have shown that the interplay of CK1 and CDK5 is necessary for efficient phosphorylation by both kinases. A phospho-mimetic mutant on the phosphorylation site of one kinase increased the phosphorylation level by the second kinase. Supporting evidence is the finding that roscovitine, a selective CDK5 inhibitor, reduced the phosphorylation of parkin by CK1 resulting from inhibition of S121 phosphorylation (Meijer et al., 1997; Rubio de la Torre et al., 2009). In further experiments, the influence of parkin phosphorylation on its activity and its effect on the formation of inclusions was examined. The results indicate that the phospho-mimetic mutant for compound phosphorylation possesses slightly enhanced enzymatic activity and showed a significantly higher tendency for aggregation (Rubio de la Torre et al., 2009).

### Huntingtin

Huntingtin is a ubiquitously expressed protein with a molecular weight of 350 kDa. It possesses a poly-Glu sequence at the N-terminus containing up to 35 CAG repeats in wild-type, whereas HD patients carry 36 or more repeats (Rubinsztein et al., 1996). There is evidence for an inverse correlation between the age of onset of symptoms and the number of CAG repeats (Andrew et al., 1993). In 65%–71% of cases, larger CAG repeats led to earlier ages of onset. Genetic and environmental factors are also playing an important role in the age of onset. Huntingtin is localized in the cytoplasm, partially in the nucleus. Its nuclear localization sequence is also found in the N-terminus and in the C-terminus a nuclear export sequence is found (Xia et al., 2003; Desmond et al., 2012). The prolongated poly-Glu sequence in HD patients inhibits the interaction between the N-terminus with the nuclear pore protein translocated promotor region which is involved in nuclear export. As a result, huntingtin is accumulated in the nucleus (Cornett et al., 2005). Noteworthy, toxic fragments of huntingtin present in the nucleus are mainly from the mutated protein due to their higher concentration in the nucleus than in the cytoplasm (Hackam et al., 1998; Lunkes et al., 2002). One therapeutic strategy is the inhibition of the formation of these fragments by modification of huntingtin (e.g., phosphorylation) to prevent the cleavage of the protein (Atwal et al., 2011).

### Ataxin-3

Ataxin-3 is a ubiquitin protease involved in transcriptional regulation and the disease protein in spinocerebellar ataxia type 3 (Burnett et al., 2003; Evert et al., 2006). It possesses nuclear and cytoplasmic functions. Its subcellular distribution is regulated through phosphorylation. As in the case of huntingtin, the nuclear presence of ataxin-3 represents a key element in the accumulation of toxic fragments (Bichelmeier et al., 2007). Analysis of 15 putative serine phosphorylation site mutants revealed that S236 in the first ubiquitin-interacting motif (UIM), S256 and S260/261 in the second UIM, as well as S340 and S352 in the third UIM, are the major phosphorylation sites of CK2 (Mueller et al., 2009). Phosphorylation of those serines determines the subcellular location of ataxin-3. Modulation of S340, S352, and S236 increases the nuclear presence of ataxin-3, while phosphorylation of S256 and S260/261 provides preferential cytoplasmic localization (Mueller et al., 2009). Apart from the influence on the cellular distribution of ataxin-3, phosphorylation plays also an important role in the solubility of the protein. As shown *in vivo* experiments phospho-mimetic mutants formed aggregates in the nucleus (Mueller et al., 2009). The effect of two selective CK2 inhibitors (DMAT and TBB) on the localization and presence of inclusions was examined in cell culture (Pagano et al., 2008). Inhibition of CK2 resulted in lower nuclear localization of ataxin-3 and less formation of protein aggregates (Mueller et al., 2009).

### Presenilin-2

*PSEN1* and *PSEN2* genes, containing 10 exons, encode presenilin-1 and presenilin-2, respectively, which play important roles in AD pathogenesis. Presenilin is a part of the γ-secretase complex responsible for the cleavage of APP to generate Aβ peptides. Mutations in *PSEN1/2* and deletions leading to alternative transcripts are associated with AD and FTD (Raux et al., 2000; Evin et al., 2002; Marcon et al., 2009). Incorrect transcripts, like *PS2V* lacking exon 6, are related to different diseases, e.g., AD (Braggin et al., 2019). *PS2V* produces truncated presenilin-2 containing 124 amino acids and only 1 of 9 transmembrane domains. This isoform was identified in the brain of AD patients with elevated levels leading to an increased amount of Aβ peptide (Sato et al., 1999; Smith et al., 2004). Two phosphorylation sites for CK2 (S7 and S9) and one for CK1 (S19) were identified (Walter et al., 1996). S19 phosphorylation elevated the binding of AP-1 protein to presenilin-2, whereas S7 and S9 phosphorylation did not show any change in the binding of activator protein 1 (Sannerud et al., 2016). Further experiments are necessary to examine the role of phosphorylation in the case of presenilin-2.

### Inhibitor-2 PP2A

The phosphorylation of tau is regulated by phosphoseryl/phosphothreonyl protein phosphatase PP2A and its activity is decreased in AD brain. PP2A is regulated by two endogenous inhibitory proteins called I_1_^PP2A^ and I_2_^PP2A^ (Gong et al., 1993; Li et al., 1996). Typically, I_2_^PP2A^ is mainly located in the nucleus regulating DNA replication, gene transcription, cell-cycle progression, DNA repair and migration as well as chromatin remodeling. In AD patients, I_2_^PP2A^ is overexpressed and translocated from the nucleus into the cytoplasm, where PP2A and significantly hyperphosphorylated tau is localized forming the NFTs in the neuronal cytoplasm (Tanimukai et al., 2005). In PC12 cells, stably transfected with tau and transiently transfected with human I_2_^PP2A^, accumulation of the inhibitor in the cytoplasm was observed (Chohan et al., 2006). CK2 was identified as one of two kinases responsible for the phosphorylation of S9 (Vera et al., 2007). This phosphorylation affects the ability of I_2_^PP2A^ to bind to importin proteins (importin-α and importin-β). Phosphorylated I_2_^PP2A^ does not form a complex with importin, therefore, it is localized in the cytoplasm instead to be transported into the nucleus (Yu et al., 2013).

## Kinase inhibitors used in NDD pathway interrogation

In the literature synthetic and natural substances are described inhibiting protein kinases CK1, TTBK, and CK2. Table 2 gives an overview of several inhibitors with *in vitro* and/or *in vivo* activities on these kinase targets. Many of them do not selectively inhibit kinases, but might be a good starting point for drug design. Many research results on cancers and cancer cell lines involving protein kinase inhibitors were published, but only a few reports towards NDDs, especially in the case of CK2. Cancer and NDDs are both characterized by the dysregulation of the same signalling pathways, but with opposite effects. In cancers the cell survival and proliferation is increased, whereas, in NDDs those alterations lead to cell death and apoptosis. The most altered signal pathways in cancer, e.g., Nrf2 pathway and Wnt/β-catenin pathway, are also implicated in NDDs, like AD and PD (Varela and Garcia-Rendueles, 2022).

**TABLE 2:** Inhibitors of CK1, TTBK, and CK2.

Inhibitor name and structure	Biological activity	References
**CK1 inhibitors**		
	- heterocyclic, central nervous system (CNS)-penetrating, and ATP-competitive inhibitors of CK1δ (IC_50_ values of 23 nM and 47 nM)	Alquezar et al. (2016); Martínez-González et al. (2020); Morales-Garcia et al. (2017); Posa et al. (2019); Salado et al. (2014)
. 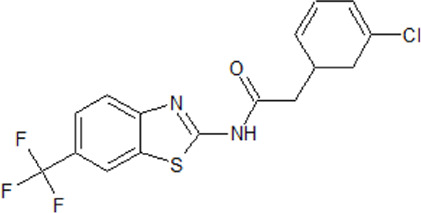	- selective over a 456 kinases panel	
	- decrease of TDP-43 phosphorylation in cell culture assays	
IGS-2.7	- able to prolongate the *Drosophila* lifespan by inhibition of TDP-43 neurotoxicity	
. 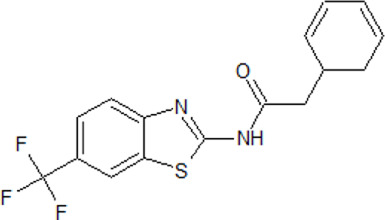	- IGS-2.7 possesses protective activity on dopaminergic neurons induced by 6-hydroxy dopamine (6-OHDA) and reduces the lipopolysaccharide inflammatory activation in primary cell cultures of astrocytes and microglia	
IGS-3.27	- effect on cell proliferation, TDP-43 phosphorylation, and subcellular localization	
	- effect on TDP-43 dependent repression of *CDK6* expression	
	- progranulin-deficient cells treated with 5 μM IGS-2.7 led to a potent inhibition of TDP-43 phosphorylation and normalization of the abnormal cytosolic TDP-43 accumulation	
	- expression of *CDK6* mRNA and the amount of TDP-43 was decreased by IGS-2.7	
	- IGS-2.7 prevented cytosolic TDP-43 accumulation in a human neuroblastoma SH-SY5Y cell model through CK1δ inhibition	
	- able to reduce TDP-43 phosphorylation in human cells derived from FTD and ALS patients	
	- IGS-2.7 is active in a TDP-43 transgenic mouse (A315T) model and in a human cell-based model of ALS	
. 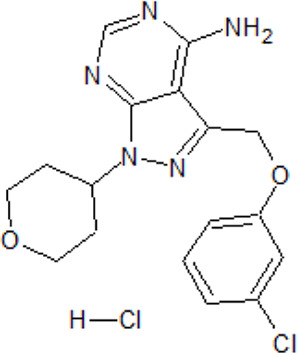 PF-4800567	- selective ATP-competitive CK1 ε inhibitor- higher inhibitory activity towards CK1 ε than towards CK1δ with IC_50_ values of 32 and 711 nM as well as IC_50_ values of 2.65 and 20.38 μΜ in whole cells, respectively- blocks period protein 3 (PER3) nuclear localization mediated by CK1 ε (0.01-10 μM ) and suppresses PER2 degradation at μΜ - rapid absorption and distribution in the plasma and brain of mice- extension of the period for single phases of the molecular clockwork, especially the duration of PER2-mediated transcriptional feedback	Meng et al. (2010); Walton et al. (2009)
. 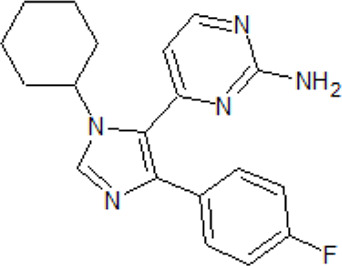	- effective and selective inhibitor of CK1ε and CK1δ (IC_50_ values of 7.7 nM and 14 nM, respectively)	Adler et al. (2019); Badura et al. (2007); Cheong et al. (2011); Sundaram et al. (2019)
PF-670462	- influence on the localization of the GFP signal back to the cytoplasm dependent on the inhibitor concentration, with an EC_50_ of 290±39 nM in CKIε-transfected COS7 cells	
	- a potent inhibitor of the Wnt/β-catenin signaling pathway with an IC_50_ of ∼17 nM	
	- weak inhibition of cell proliferation and only moderately inhibition of HEK293 and HT1080 cell growth (1 μM)	
	- potential to repeal hippocampal proteomic changes in several AD-related and clock-regulated pathways, e.g., synaptic plasticity and APP cleavage	
	- able to reverse effects of working memory deficits and lead to the improvement of disturbances in behavioral circadian rhythm	
	- inhibition of CK1δ/ε increases the cognitive-affective behavior and inhibits the amyloid amount in the APP-PS1 mouse model of AD	
. 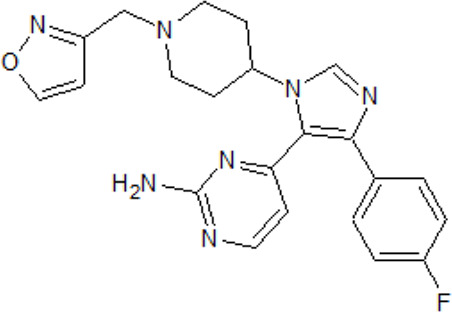 PF-5006739	- possesses strong and selective potency against CK1δ and CK1ɛ in enzymatic assays (3.9 nM and 17 nM, respectively) and in whole-cell screening (EC_50_ = 15.2 and 83 nM, respectively)- in a panel of 59 kinases, only JNK2 and MAP4K6 are inhibited at a concentration of 1 µM with IC_50_ values of 6.1 and 1.5 µM, respectively- used in the treatment of several psychiatric disorders- lowers the effect on opioid drug-seeking behavior in a rodent operant reinstatement animal model dependent on the inhibitor dose- daily treatment of diet-induced obese and ob/ob mice increase expression of clock genes and improved the glucose tolerance	Cunningham et al. (2016); Wager et al. (2014)
. 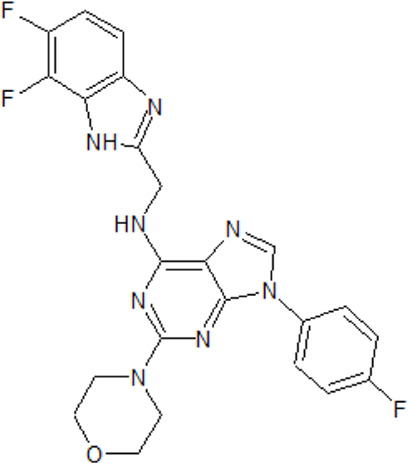	**-** effective ATP-competitive CK1 inhibitor (IC_50_ values of 44 nM for CK1δ and 260 nM for CK1ε)- EC_50_ value below 100 nM estimated in a MTT assay against human melanoma cell line A375- a binding assay analysis of 442 kinases showed that only CK1δ and CK1ε are strongly inhibited- reduction of the activities of CDK6/cyclin D3, CDK6/cyclin D1, CDK4/cyclin D3, CDK4/cyclin D1, and FLT3 (IC_50_ values between 368 and 3,000 nM)- decrease of TPA-induced skin tumor formation in carcinogen-initiated mouse skin cells, most likely by the inhibition of the Wnt/β-catenin signaling	Bibian et al. (2013); Su et al. (2018)
SR-3029
. 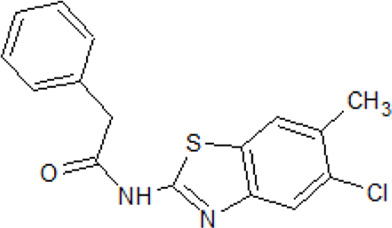 LH846	**-** selective, cell-permeable, ATP-site-targeting inhibitor (IC_50_ values of 290 nM, 1.3 μM and 2.5 μMfor CK1δ, ε , and α,respectively) - inhibition of PER1 phosphorylation by CK1δ and its degradation- able to prolongate the circadian period in U2OS cells, but only minimally effects the amplitude	Lee et al. (2011)
. 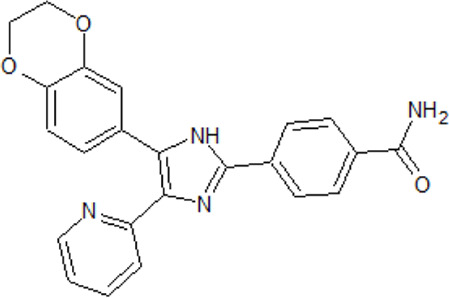	- effective, reversible, and weakly specific ATP-competitive inhibitor of CK1δ and ALK5 with IC_50_ values of 0.3 and 0.5 µM, respectively	Flajolet et al. (2007); Järås et al. (2014); Liu et al. (2021); Rena et al. (2004)
D4476	- modest inhibitory activity against other kinases, including p38α MAP kinase, PKB, SGK, and GSK-3β- potently kills leukemia stem cells (LSCs) with high selectivity when compared to normal HSPCs- CK1α inhibition causes a decrease of ribosomal protein S6 phosphorylation and activates p53 resulting in the selective removal of leukemia cells	
	- inhibition or down-regulation of CK1α, efficiently reduced glioblastoma multiforme (GBM) cell proliferation in both Tp53 wild-type and Tp53-mutant GBM cells	
	- significant reduction of Aβ40 peptide production in N2A cells expressing APP-695	
	- effect towards γ-secretase cleavage activity in mammalian cells transfected with the C-terminal fragment of APP
. 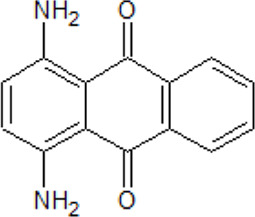	**-** compound 1: ATP-competitive and isoform CK1δ seletive inhibitor (K_i_ = 125 nM)	Cozza et al. (2008)
Amino-antraquinone 1	- compound 2: inhibitory activity against CK1δ (IC_50_ = 0.6 μM)- cytotoxicity of compound 1 on human ovarian carcinoma cell line 2008 (IC_50_ = 14.4 μM) and on its cisplatin-resistant clone C13 ( IC_50_ = 87.9 μM)- cytotoxicity of compound 2 on human ovarian carcinoma cell line 2008 (IC_50_ = 122.4 μM) and on its cisplatin-resistant clone C13 ( IC_50_ = 8.0 μM)	
. 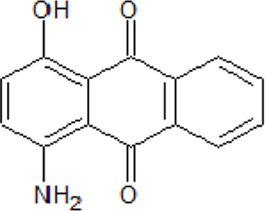		
Amino-antraquinone 2		
. 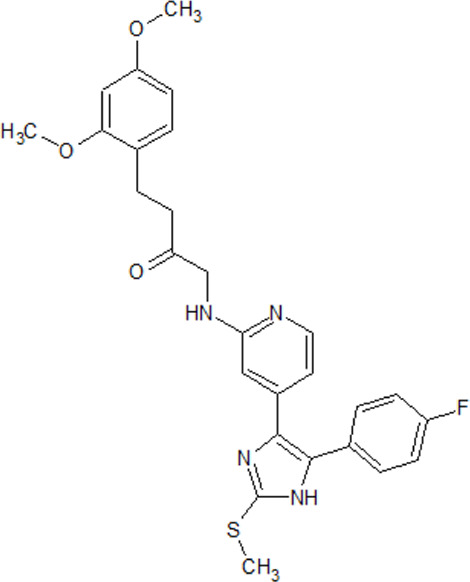	- highly effective ATP-competitive inhibitor of CK1δ (IC_50_ CK1δ=4 nM, IC_50_ CK1ε=25 nM)- highly selective towards CK1δ when tested against more than 321 protein kinases- high efficiency against p38α MAPK with an IC_50_ value three-fold higher compared to CK1δ- inhibitory effect on human pancreatic cancer cell lines Colo357 and Panc89 (EC_50_ of 3.5 and 1.5 μM, respectively)	Halekotte et al. (2017)
Compound 11b
. 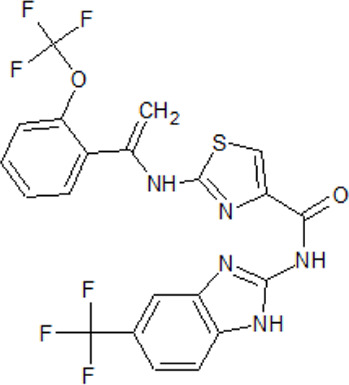	- potent ATP-competitive and specific inhibitors of CK1δ (IC_50_ values of 40 and 42 nM, respectively)	Bischof et al. (2012)
Bischof-5	- **Bischof**-**5** exhibits a 5-fold higher affinity towards CK1δ than to CK1ε **(**IC_50_=199 nM)	
	- **Bischof**-**6** inhibits both isoforms in similar range (IC_50_=33 nM for CK1ε)	
. 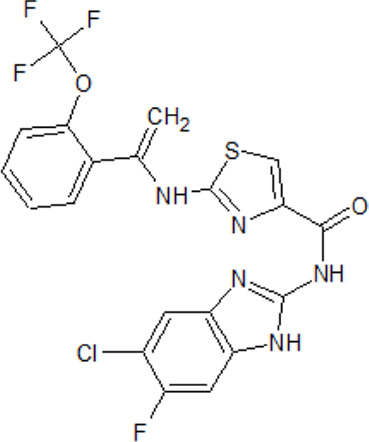	- **Bischof-5** is highly potent and selective towards CK1δ in a panel of 442 kinases	
Bischof-6	- Bischof 5 and 6 negatively influence the proliferation of several tumor cell lines	
. 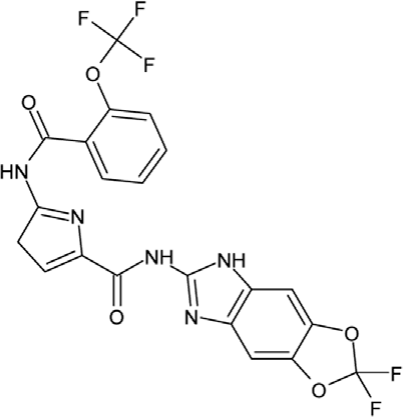	- potent ATP-competitive and selective CK1δ/ε inhibitors (IC_50_ values of 0.07-0.81 μM and 0.13-1.36 μM)	Richter et al. (2014)
Compound 1		
. 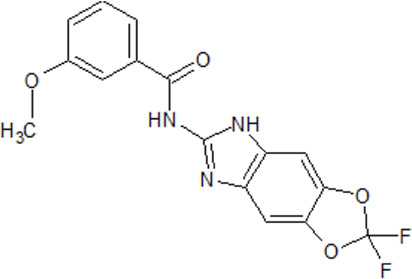	- inhibition of growth analysed by cell viability assays and cell cycle distribution on 82 different tumor cell lines	
Compound 2		
. 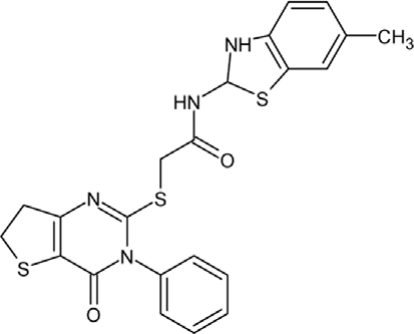	- ATP-competitive and CK1 specific	García-Reyes et al. (2018)
IWP-2	**-** specifically inhibits CK1δ when compared to 320 other kinases (IC_50_ values of 0.317 and 4 μM for CK1δ and CK1ε, respectively- inhibition of the gatekeeper mutant^M82F^CK1δ (IC_50_ = 40 nM)- inhibition of the viability of various cancer cell lines	
. 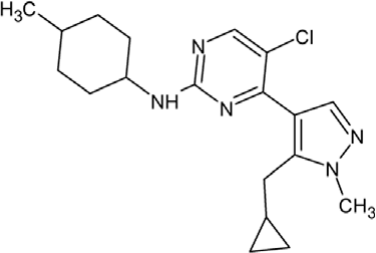	- multi-kinase inhibitor (IC_50_ values between 0.5 and 20 nM for CK1 isoforms, CDK7, and CDK9)	Ball et al. (2020)
BTX-A51	- specifically blocks leukemic stem cell target CK1α as well as CDK7 and CDK9 preventing transcription of key oncogenic genes.	
	- activation of p53 and its sustained stabilization by a super-enhancer shutdown of Mdm2 in combination with the transcriptional shutdown of leukemia oncogenes, including *Myc* and *Mcl1*	
. 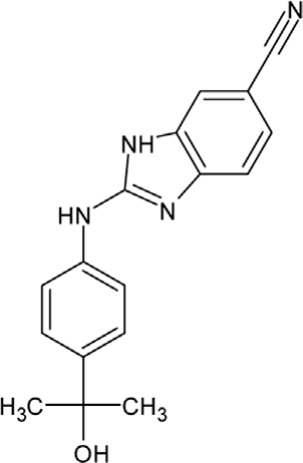	- highly selective and CK1 isoform-specific (IC_50_ = 14 nM for CK1γ)	Hua et al. (2012)
Compound 1h	- excellent selectivity over other CK1 isoforms, like CK1α (IC_50_ = 9.18 μM) and CK1δ (IC_50_ = 2.32 μM)	
	- no inhibitory activity against 48 kinases including GSK3β (IC_50_ = 60 μM)	
	- stable in the rat and human microsomes and show good effects on cells and modest pharmacokinetic properties in rats	
. 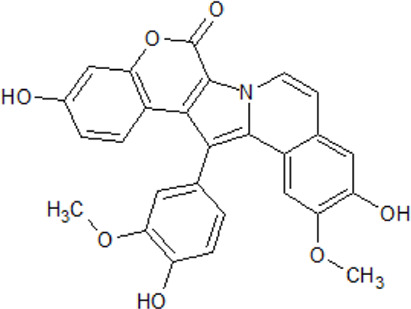	- inhibitory activity against CK1δ and CK1ε (IC_50_ values of 0.41 and 0.8 μM, respectively)	Baunbæk et al. (2008); Iwao et al. (2011)
Lamellarin 3	- inhibition of other kinases (CDKs, DYRK1A, GSK3α/β, and PIM1) with IC_50_ values from 0.06–0.6 μM	
. 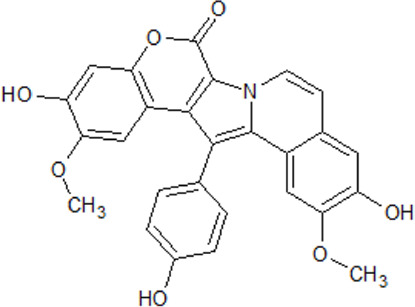	- inhibition of cell survival of human neuroblastoma SH-SY5Y cells (IC_50_ values of 0.56 and 0.11 μM for lamellarin 3 and 6, respectively)	
Lamellarin 6		
. 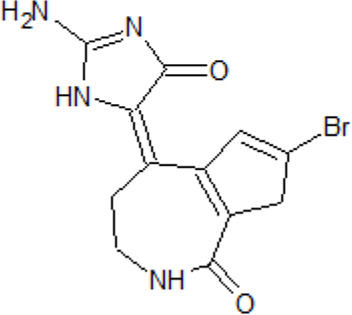	- inhibitory activity in nanomolar range against few AD-related protein kinases, e.g., CK1δ, GSK3β, and CDK5/p25 (IC_50_ values of 35, 10, and 28 nM, respectively)	Meijer et al. (2000); Plisson et al. (2014); Wan et al. (2004); Zhang et al. (2012)
Hymenialdisine	- numerous kinases were inhibited only in the micromolar range, e.g., Aurora-A, Her1/2, IKKα, PKA, and PKB- CK1 dose-dependent inhibition of presenilin-2 phosphorylation using presenilin-2-maltose-binding protein- debromohymenialdisine inhibits the activities of diverse protein kinases including CK1δ, CDK5/p25, and GSK3β (IC_50_ values of 0.1–0.4 μM	
**TTBK1/2 inhibitors**		
. 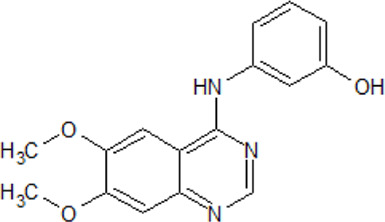	**-** potent and selective ATP-competitive inhibitors (IC_50_ values of 4.4 µM and 6.8 µM (AZ-1), 2.6 µM and 3.2 µM (AZ-2) for TTBK1 and TTBK2, respectively)	Xue et al. (2013); Kiefer et al. (2014); Palomo et al. (2020)
AZ-1	- neuroprotective profile on phospho-TDP-43 induced cell death in cellular human neuroblastoma models	
. 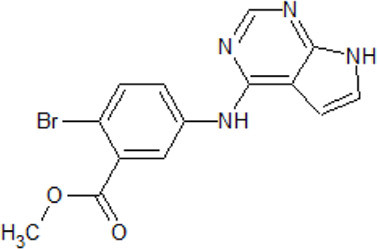		
AZ-2	
. 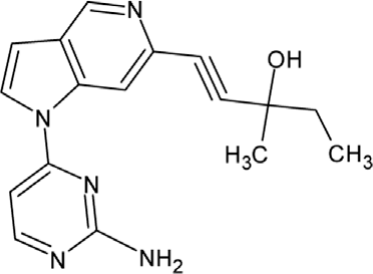	- potent, selective and brain-penetrant TTBK1 inhibitor (IC_50_ = 2.7 nM)	Dillon et al. (2020); Halkina et al. (2021); Tian et al. (2021)
TTBK1-IN-1	- in *in vivo* selectivity study with a panel of 150 kinases only 4 kinases (including TTBK1/2) are inhibited more than 50%- dose-dependent inhibition of tau phosphorylation levels at Ser422 (IC_50_ of ∼9.5 nM) in isoflurane-induced hypothermia mice model- reduction of TDP-43 phosphorylation and formation of high molecular species in N2a cells	
. 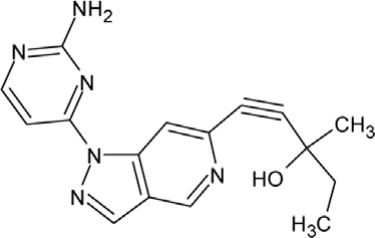	- potent, selective and brain penetrant inhibitors of TTBK1 (IC_50_ of 60 nM (compound 8) and 2.7 nM (compound 31))	Halkina et al. (2021)
Compound 8	- compound 31 inhibits tau phosphorylation at S422 in mouse hypothermia and a rat developmental model (IC_50_ of 315 nM)	
. 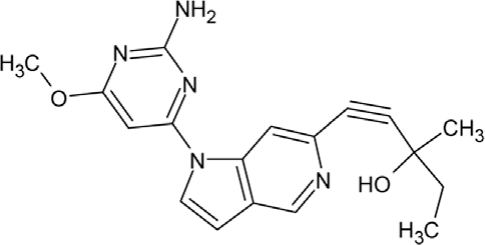	
Compound 31	
. 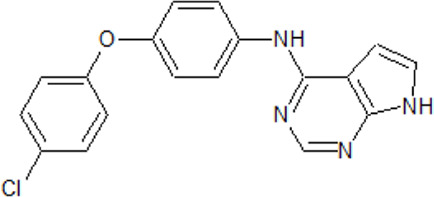	- cell-permeable, ATP-competitive TTBK1/2 inhibitor (IC_50_ values of 0.24 μM and 4.2 μM for TTBK1 and TTBK2, respectively)	Nozal et al., (2022)
Pyrropirimidine 29	- inhibition of TDP-43 phosphorylation *in vitro* and *in vivo*, in cell cultures and in the spinal cord of transgenic TDP-43 mice	
. 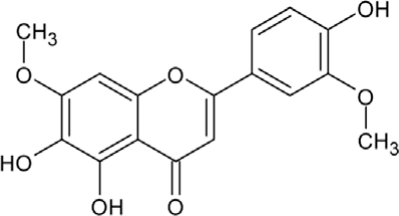	**-** possesses good CNS penetrating properties and potent antioxidant and anti-inflammatory activities	Jana and Singh, (2020); Wang et al. (2016)
5-TDMF	- inhibition of LPS-induced NF-κB translocation and expression of iNOS and COX-2 blocking MAP kinase and Erk signaling pathways	
. 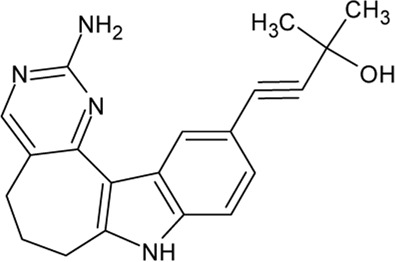	- inhibition of TTBK1 and TTBK2 (1 μM) with remaining activity of 8% and 12% (MRC Kinase Profiling Inhibitor Database)	Potjewyd et al. (2022)
AMG-28	- inhibition of tau phosphorylation at S422 in a biochemical and cellular assay with IC_50_ values of 199 nM and 1.85 μM, respectively	
	- new analogs are more potent inhibitors of TTBK2 than TTBK1	
	- assayed in NanoBRET test in permeabilized HEK293 cells and compound 9 shows IC_50_ values of 2.5 and 1.8 μM for TTBK1 and TTBK2, respectively- in an enzymatic test derivative 9 possesses inhibitory activity (IC_50_ values between 150 and 400 nM)- derivative 9 shows highest kinome-wide selectivity towards the TTBK activities	
**CK2 inhibitors**		
. 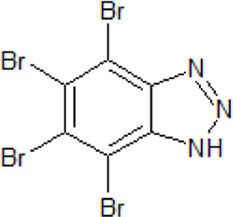	- cell-permeable, highly selective, and ATP/GTP-competitive inhibitor of CK2 (IC_50_ values of 0.9 and 1.6 μM for rat liver and human recombinant CK2, respectively)	Pagano et al. (2008); Sarno et al. (2001); Szyszka et al. (1995); Yadikar et al. (2020); Zhang et al. (2018)
TBB	- discrimination between CK2 subunits (K_i_ values ranging from 80 nM to 210 nM)	
	- strong inhibition of several other kinases (DYRK1-3, HIPK2, and PIM1–3) at a concentration of 10 μM	
	- effect on human prostate cancer PC-3 cell viability is dependent on the time of administration	
	- inhibition of okadaic acid-induced monomeric and oligomeric phospho-tau in both, N2a and CTX culture	
	- prevention of I_2_^PP2A^ phosphorylation at Ser9 in neurons and animal models	
. 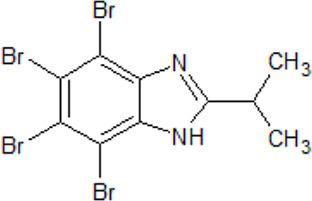	- ATP-competitive CK2 inhibitor (IC_50_ = 130 nM)	Li et al. (2011); Ławnicka et al. (2010); Pagano et al. (2008); Yde et al. (2007)
DMAT	- inhibition of PIM1-3, HIPK2-3, DYRK1-3, PKD1, and CDK2 (IC_50_ values between 0.07–3.7 µM)	
	- possesses anti-neoplastic effect on the growth and hormonal activity of human adrenocortical carcinoma cell line (H295R) *in vitro*	
	- able to induce cell death in antiestrogen-resistant human breast cancer cells	
	- inhibition of TDP-43 phosphorylation which is necessary for the decrease of the ND251 or ND207 aggregation	
. 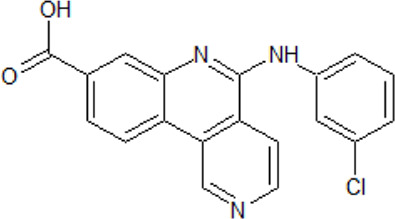	- orally bioavailable, highly selective, and potent CK2 inhibitor (IC_50_ value of 13 nM against CK2α and CK2α')	Buontempo et al. (2016); Chon et al. (2015); Cozza et al. (2012); Lee et al. (2019); Kim et al. (2014); Pierre et al. (2011); Rosenberger et al. (2016); Siddiqui-Jain et al. (2010);
Simitasertib (CX4945)	- in cancer cells, causes cell-cycle arrest and selectively induces apoptosis when compared to normal cells.	
	- correlation between the antiproliferative activity and the expression of CK2α as well as inhibition of the PI3K/Akt signaling	
	- synergistic effects on cell death in combination with other inhibitors	
	- synergistic cytotoxic effects of bortezomib (20S proteasome inhibitor with K_i_ of 0.6 nM) and CX-4945 in acute lymphoblastic leukemia resulting in turning off the prosurvival ER chaperone BIP/Grp78 and turning on the pro-apoptotic NF-κB	
	- dose-dependent inhibition of the IL-1β/TNF-α induced secretion of the inflammatory cytokines MCP-1 and IL-6 in human primary astrocytes and U373 astrocytoma cells	
	- strong inhibition of CLK activity	
	- inhibition of CDC2-like kinases in nanomolar range leading to the inhibition of the phosphorylation of serine/arginine-rich proteins in mammalian cells	
	- induction of abnormal alternative splicing of CK2α′ pre-mRNA	
	- orphan drug status by the US FDA for therapy of hard-to-treat bile duct cancers, known as cholangiocarcinomas	
	- first described orally bioavailable CK2 inhibitor that advanced into clinical trials	
. 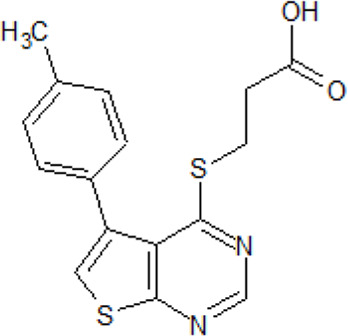 TTP22	- inhibition of CK2 with a K_i_ of 40 nM- high selectivity towards CK2 confirmed using serine/threonine (ASK1, JNK3, Aurora A, and Rock 1) and tyrosine protein kinases (FGFR1, Met, and Tie2)- inhibition of CK2 decreases taspase1-dependent MLL1 processing leading to higher MLL1 stability, and finally displace the MLL chimeras from chromatin- suppression of CK2 retard the leukemic progression in a MLL-AF9 leukemia mouse model	Golub et al. (2011); Zhao et al. (2018)
. 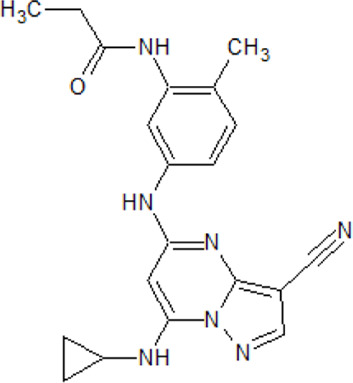 SGC-CK2-1	**-** potent inhibitor of CK2 with activity on both isoforms, CK2α and CK2α’ (IC_50_ values of 4.2 nM and 2.3 nM, respectively)- inhibition of DYRK2 (IC_50_ of 440 nM)- potent suppression of CK2-mediated neuroinflammatory response inhibiting the expression of the proinflammatory cytokines IL-6 and IL-1β	Wells et al. (2021); Mishra et al. (2022)
. 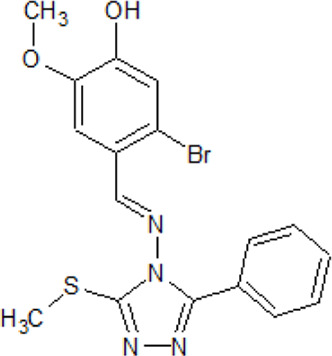 GO289	**-** potent and selective inhibitor of CK2 (IC_50_ of 7 nM) and minor effects on CK1δ and CK1α activities- inhibition of PIM2 (IC_50_ of 13 nM)- inhibition of the phosphorylation of clock proteins, including PER2- cell type-dependent inhibition of cancer cell growth that correlated with cellular clock function- *in vitro* potency and selectivity comparable to CX4945	Borgo et al. (2021b); Oshima et al. (2019)
. 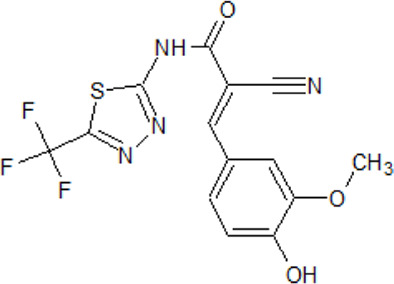	- potent and selective dual inhibitor of CK2 and serine-arginine protein kinase 1 (SRPK1)	Dalle Vedove et al. (2020); Morooka et al. (2015)
SRPIN803-rev	- activity in mouse model of age-related macular degeneration due to the involvement of SRPK1 in angiogenesis and CK2 in neovascularization	
. 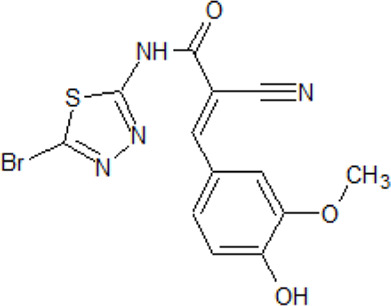 Compound 4	**-** the most potent SRPIN803-rev derivative (IC_50_ = 0.28 μM)- significant selectivity when tested on 320 kinases (inhibits only CK2 catalytic subunits by more than 50% at 1 μM concentration)- good cell permeability, inhibiting endocellularly CK2- significant reduction of Jurkat and CEM cells viability	Dalle Vedove et al. (2020)
. 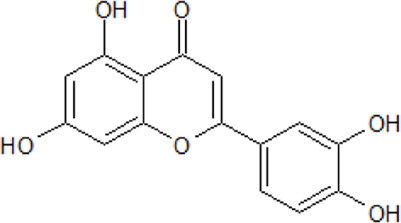	**-** antioxidant, anti-cancer, and anti-inflammatory activities	Caltagirone et al. (2015); Daily et al. (2021); Guo et al. (2013); Kang et al. (2004); Kotanidou et al. (2002); Kwon, (2017); Lolli et al. (2012); Lopez-Lazaro, (2009); Rezai-Zadeh et al. (2009); Sharma et al. (2007)
Luteolin	- ATP-competitive inhibition of CK1 (IC_50_ = 1.6 μM)	
	- inhibition of CK2 holoenzyme and CK2α (IC_50_ 0.5 and 0.35 μM, respectively)	
	- prevention of NDDs by reducing oxidative stress, inflammation, and Aβ production	
	- protection against lipopolysaccharide-induced lethal toxicity and a reduction of the expression of pro-inflammatory intermediate in mice (0.2 mg/kg)	
	- reduction of 6-hydroxy-dopamine-derived toxicity leading to neuroprotection in rat pheochromocytoma PC12 cells (3.13–50 μM)	
	- neuroprotective effects on stroke patients undergoing neurorehabilitation as a component of a co-ultramicronized composite (140 mg/day for 60 days) and palmitoylethanolamine	
	- improvement of brain insulin resistance as well as inflammation protecting against the development of AD and the gut microbiota-liver-brain axis	
. 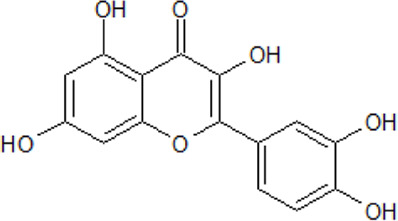 Quercetin	- antioxidant and anti-inflammatory activities- potent inhibition of all CK2 isoforms with IC_50_ values below 1 µM- affects ROS-producing enzymes and protects neurons from oxidative stress-induced damage- potential up- and/or down-regulation of cytokines *via* Nrf2, ERK1/2, PI3K/Akt, JNK, MAPK pathways- Improvement of cognitive performance and cognitive functions in patients with neurological diseases or neurobehavioral disorders- inhibition of Aβ production *in vitro* and protection against cognitive impairments in a mouse model	Baier et al. (2018); Nakagawa and Ohta, (2019); Selvakumar et al. (2012); Zaplatic et al. (2019)
. 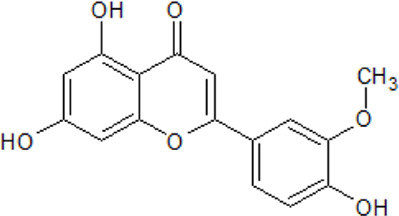 Chrysoeriol	- inhibition with IC_50_ values in low nanomolar range- administration alleviates the damage caused by cerebral ischemia and reperfusion in MCAO model rats- administration reduces the area of brain infarction and relief of neurobehavioral deficits- inhibition of the production of pro-inflammatory cytokines,- reduction of neuronal apoptosis and promotion of nerve growth- neuroprotective mechanism is strongly linked to the activation of the Wnt/β-catenin signaling pathway	Baier et al. (2017); Shao et al. (2021)
. 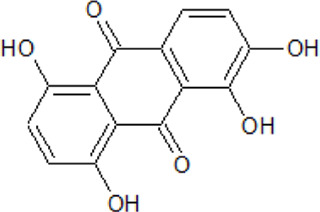	- potent, selective, ATP-competitive, and cell-permeable inhibitor of CK2 ( K_i_ = 50 nM)	Cozza et al. (2009); Cozza et al. 2015; Schwind et al. (2017); Zhou et al. (2015)
**Quinalizarin**	- able to discriminate between the free catalytic subunits and the CK2 holoenzymes	
	- downregulation of transcription factors and modulation of microRNA in 3T3-L1 cells leading to inhibition of adipogenesis	
	- inhibition of cell viability, especially in adenocarcinoma cells harboring EGFR sensitive mutation and interruption of migration	
	- stimulation of apoptosis in different human lung cancer cell lines	
. 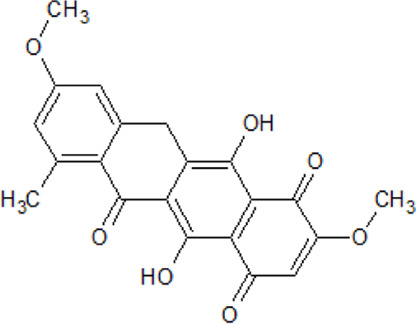 Bikaverin	- moderate ATP-directed inhibitor of the CK2 holoenzyme (IC_50_ of 1.24 µM)- reduction of cell viability and proliferation in cancer cell lines, like MCF7, A427, and A431 (10 μM)TTBK1/2 inhibitors- cytotoxic and antitumor effect on L5178Y lymphoma cells (IC_50_ of 0.23 μg/ml) and on BALB/c mice inoculated with L5178Y- capable to recover the nuclear and mitochondrial integrity in H_2_O_2_-induced damaged cells	Haidar et al. (2019); Nirmaladevi et al. (2014); Hinojosa-Ventura et al. (2019)
. 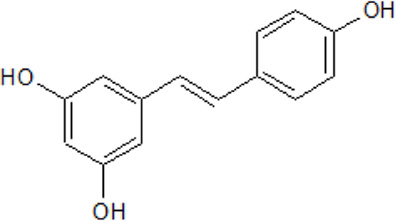 Resveratrol	**-** antioxidant, anti-cancer, anti-inflammatory, and anti-aging properties- CNS penetrating molecule- increases the activity of antioxidant enzymes- reduction of the cell viability of human breast carcinoma cells (MCF-7) dependent on the concentration (IC_50_ = 106 μM)- inhibition of CK2 activity by 1.6-fold- decrease of the potential of the mitochondrial membrane- increases ROS levels by 1.7-fold- protection of the central nervous system against symptoms of disorders, like stroke, spinal cord injury-induced inflammation, AD, PD, or HD- activation of SIRT1, Nrf2, and AMP-activated kinase	Ahmed et al. (2017); Costa et al. (2021); Gomes et al. (2018); Farkhondeh et al. (2020); Kim et al. (2018); Komorowska et al. (2020); Kumar et al. (2011); Turner et al. (2015); Liu et al. (2015); Zhao et al. (2021); Maher et al. (2010); Yu et al. (2016); Su et al. (2021); Yang et al. (2021)
. 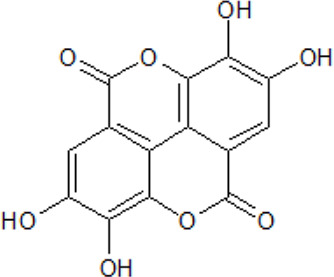	**-** potent ATP-competitive CK2 inhibitor (IC_50_ = 40 nM, K_i_ = 20 nM)	Baluchnejadmojarad et al. (2017); Cozza et al. (2006); Dheen et al. (2005); Ebrahimi et al. (2019); Lastres-Becker et al. (2016); Liu et al. (2017); Ríos et al. (2018); Vargas et al. (2006); Wang et al. (1998); Wei et al. (2020)
Ellagic acid	- inhibitory effects towards the activity of many kinases such as LYN, PKA, SYK, GSK3, PKC, FGR, or CK1 (IC_50_ values between 2.9 and 13 µM)	
	- normalization of the lipid metabolism and the lipidemic profile	
	- regulation of proinflammatory mediators, such as IL-6, IL-1β, and TNF-α	
	- upregulation of Nrf2 and the inhibition NF-κB	
	- neuroprotection due to antioxidant properties, ability to iron chelating, the induction of signaling pathways, and the reduction of mitochondrial dysfunction	
	- neuroprotective contribution towards several neurotoxins in numerous animal models	
	- neuroprotective effect in the 6-OHDA rat model of PD (50 mg/kg/day for one week)	
	- neuroprotective potential by the reduction of apoptosis and oxidative stress, and inhibition of MAO-B.	
	- effect on the ERβ/Nrf2/HO-1 signaling cascade	
	- protective influence on DA neurons from rotenone-induced neuronal damage by activating Nrf2 signaling	
	- induction of Nrf2 and HO-1 expression and inhibition of the NF-κB signaling pathway	
	- protection of rats (6-OHDA rat model) against MTX-induced apoptosis and mitochondrial dysfunction	
	- significant reduction of the volume of cerebrum infarction and the neurological deficit scores of the rats in an experimental rat model based on oxygen-glucose deprivation and reoxygenation in primary cultured cortical rat neurons.	
	- increase of the number of Bcl-2-positive cells and the ratio of Bcl-2-positive to Bax-positive neurons in the semidarkness zone near the brain ischemic focus in the photothrombotic cerebral ischemia model	
	- higher neuron viability, cell nuclear integrity, and a higher ratio of Bcl-2/Bax expression in the primary cultured neuron model	
	- decrease of the number of apoptotic cells	

The development of specific CK1 inhibitors capable to cross the blood-brain-barrier is a promising target for the treatment of TDP-43 proteinopathies, e.g., ALS. Small brain-penetrating molecules were described which block the neurotoxicity of TDP-43 in cell culture experiments through inhibition of its phosphorylation (Perez et al., 2011; Salado et al., 2014; Morales-Garcia et al., 2017). Up to now, few CK1-specific compounds have been synthesised and a small part of them has been also examined in animal models. Kinetic studies of these compounds revealed an ATP-competitive mode of action in the case of almost all molecules.

In several studies, it has been proven that CK1 activity is necessary for molecular pacemaking. It was shown that CK1δ is the main regulatory element of the clock period: inhibition of CK1δ remarkably prolongated the circadian rhythms in locomotor activity *in vivo* and molecular oscillations in the suprachiasmatic nucleus (SCN) and peripheral tissue slices *in vitro*. Additionally, cumulation of PER2 protein in the nucleus was observed, *in vitro* and *in vivo* (Meng et al., 2010).

Cell proliferation is increased and colony formation is promoted through overexpression of CK1α. Effects of CK1α inhibition include the increase of the sensitivity to radiotherapy and reduction of the production and secretion of pro-inflammatory factors (Liu et al., 2021).

Several benzimidazole-based inhibitors displayed significant inhibition of CK1δ, e.g., Bischof-5 and -6. Other potent ATP-competitive and selective CK1δ/ε inhibitors are represented by difluoro-dioxolo-benzimidazole derivatives, compounds 1 and 2 (Richter et al., 2014). Substances derived from inhibitors of Wnt production (IWP) are structurally similar to benzimidazoles. Such inhibitors have been characterized as ATP-competitive and CK1 specific.

Another potent inhibitor, compound 1h, which is highly selective and CK1 isoform-specific was identified from a high-throughput screen of the Amgen compound library (Hua et al., 2012).

Many modulators of CK1 presently under investigation are isolated from natural environment or are derivatives of natural products. Nowadays, compounds from marine organisms are getting more attention and are now being investigated in clinical tests, essentially against cancer, inflammation, chronic pain, and NDDs. Among the promising drug candidates is the family of lamellarins, which are marine alkaloids with fused 14-phenyl-6H-[1]benzopyrano[4′,3′:4,5]pyrrolo[2,1-a]isoquinoline or non-fused 3,4-diarylpyrrole-2-carboxylate ring systems (Baunbæk et al., 2008; Bailly, 2015; Fukuda et al., 2020). So far, over 50 lamellarins have been purified from different marine organisms, e.g., mollusks, tunicates, and sponges. In 2008, protein kinases have been identified as new molecular targets of anticancer lamellarins (Baunbæk et al., 2008). 22 lamellarins were screened for cancer- and Alzheimer’s disease-relevant protein kinases.

Until now, only a few protein kinases were described, which are associated with abnormal TDP-43 hyperphosphorylation, including both TTBK isoforms (Versluys et al., 2022). Both are known to colocalize with TDP-43 inclusions in spinal cords of ALS patients. TTBK2 is involved in crucial cellular mechanisms, e.g., ciliogenesis, microtubule dynamics, and neurotransmitter trafficking. Thus, its reduced activty may have negative effects for the patients (Jackson, 2012; Bowie and Goetz, 2020). Only a small number of TTBK1/2 inhibitors have been described, but unfortunately they do not show selectivity for one isoform (Xue et al., 2013; Kiefer et al., 2014).

A set of TTBK1 azaindazole inhibitors has been examined (Halkina et al., 2021). Two of them are characterized by high potency, selectivity and they are brain penetrant: compound 8 (4-(1-(2-Aminopyrimidin-4-yl)-1*H*-pyrazolo[4,3-c]pyridin-6-yl)-2- methylbut-3-yn-2-ol) with an IC_50_ of 60 nM and compound 31 ((*S*)-1-(1-(2-Amino-6-methoxypyrimidin-4-yl)-1*H*-pyrrolo[3,2-c]-pyridin-6-yl)-3-methylpent-1-yn-3-ol) with an IC_50_ of 2.7 nM.

Lately, receptor-based pharmacophore models were developed applying three TTBK1 protein structures. The combination of integrated e-pharmacophore based virtual screening and molecular dynamics simulation resulted in four hits: ZINC14644839 (5,6,4′-Trihydroxy-7,3′-Dimethoxyflavone, 5-TDMF), ZINC00012956 (3-phenyl-2-(9H-purin-6-ylamino)propan-1-ol), ZINC91332506 (1-[3-(6-aminopurin-9-yl)propyl]-3-methyl-pyridin-2-one), and ZINC69775110 (N-[(4-ethoxy-3-fluoro-phenyl)methyl]-7H-purin-6-amine). AMG-28 (4-(2-amino-5,6,7,8-tetrahydropyrimido[4′,5′:3,4]cyclohepta[1,2-b]indol-11-yl)-2-methylbut-3-yn-2-ol) was originally designed as an inhibitor of a Ser/Thr protein kinase essential for the activation of the NF-κB pathway (NIK) (Li et al., 2013). A co-crystal structure of this inhibitor with the kinase domain of human TTBK1 showed binding of the aminopyrimidine ring with the hinge region of protein kinase. On the basis of AMG-28 11 new indolyl pyriminidine compounds were synthetized. New analogs are more potent inhibitors of TTBK2 than TTBK1 (Potjewyd et al., 2022).

Compounds that may inhibit CK2 activities where described in numerous publications. Since research results on the role of CK2 in NDD are diverse, there are only a few reports about CK2 inhibitors on NDDs available. The observation that CK2 is either overactive or overexpressed in patient brains supports kinase inhibition as a therapeutic approach for multiple neurodegenerative diseases. A large group of CK2 inhibitors, known for more than 20 years, are benzimidazoles, e.g., TBB and DMAT (Szyszka et al., 1995).

Data propose the influence of CK2 on astrocytes in the neuroinflammatory response in AD. In astrocytes in the hippocampus and temporal cortex of AD patients levels of CK2α/α’ are increased. Those astrocytes are linked to amyloid deposits in the AD brain. In human primary astrocytes and U373 astrocytoma cells, the IL-1β/TNF-α induced secretion of the inflammatory cytokines MCP-1 and IL-6 is potently inhibited by CX-4945 dependent on the dose (Rosenberger et al., 2016). CX-4945 is the first described orally bioavailable CK2 inhibitor that advanced into clinical trials (Pierre et al., 2011; Cozza et al., 2012).

Quite recently SRPIN803-rev (6-(4-hydroxy-3-metoxybenzylidene)-5-imino-2-(trifluoromethyl)-5H-[1,3,4]thiadiazolo[3,2-a]pyrimidin-7(6H)-one), a new dual inhibitor of CK2 and serine-arginine protein kinase 1, was identified (Dalle Vedove et al., 2020). SRPIN803-rev and its new synthesized derivatives bind to the open conformation of the hinge/αD region within the ATP-binding pocket of CK2α.

The MLL/COMPASS stability is regulated by taspase1 cleavage and might be a possible target for clinical therapy of leukemia. Destabilized MLL and unprocessed version MLL1 associated with chromatin results in the displacement of MLL chimeras from chromatin in leukemic cells. The CK2 phosphorylation site is next to the taspase1 cleavage site, and enables its cleavage. Inhibition of CK2 by specific inhibitors (CX-4945 or TTP22) decrease taspase1-dependent MLL1 processing, which leads to higher MLL1 stability, and finally displace the MLL chimeras from chromatin. In a MLL-AF9 leukemia mouse model the suppression of CK2 retard the leukemic progression (Zhao et al., 2018).

Naturally occurring compounds might act as antioxidant, anti-inflammatory, antiviral, antimicrobial, and anticancer agents (Baier and Szyszka, 2020). They have shown neuroprotective effects in many clinical trials (Ullah et al., 2020; Akter et al., 2021; Kim and Park, 2021; Wang et al., 2021).

It has been proven that flavonoids are most effective in the treatment of NDDs, including AD. First analyses with flavonoids against CK2 were reported by Li et al. (2009), Lolli et al. (2012). As we described in our own publications, flavonoids naturally occurring in plants are highly potent CK2 inhibitors. A set of more than 20 compounds (e.g., apigenin, pedalitin, and chrysoeriol) was tested for their inhibitory effect on four human CK2 isoforms. The results reveal that CK2α’ was most sensitive to the examined compounds (Baier et al., 2017; Baier et al., 2018).

Quercetin (3,5,7,3′,4′–pentahydroxyflavone) belongs to the polyphenolic compounds with powerful antioxidant and anti-inflammatory activities. Polyphenolic compounds are often applied in the treatment and protection against severe diseases, like diabetes, cancer, neurodegenerative and cardiovascular diseases.

In time-course experiments it was shown that CK2 is crucial at early time points just after the induction of cell differentiation (Schwind et al., 2017).

In several studies it was observed that Nrf2 signaling is involved in PD pathogenesis (Dheen et al., 2005). The increase of Nrf2 induced dopamine (DA) neuroprotection and, at the same time, the decrease of Nrf2 altered DA neurons to get sensitive to oxidative stress damage (Lastres-Becker et al., 2016). It was shown that the progress of PD is linked to an incomplete activation of Nrf2 (Vargas et al., 2006).

## Conclusion

During past decades many research groups provided new information to better understand the molecular aspects of cancerogenesis and neurodegenerative diseases. Protein kinases play an important role in the regulation of the activity of a huge amount of proteins involved in the control of different cell functions. Nevertheless, in many cases of NDDs, protein aggregation often caused by (hyper-)phosphorylation is observed. Therefore, the inhibition of these reactions is a promising therapeutic target. Unfortunately, whereas for the treatment of cancers several compounds were successfully developed, there does not exist a therapy for NDDs being a kinase inhibitor. Until September 2021, 73 small molecule kinase inhibitors were approved by FDA but only a small amount of them are for noncancer-related diseases (Ayala-Aguilera et al., 2022). The main obstacle in the design of substances targeting the CNS is the effective crossing of the blood-brain-barrier which is necessary in the treatment of NDDs, but also in the case of oncology. CK1 superfamily and CK2 play essential roles in the regulation of cell processes, like in signaling pathways. With respect to this fact, it is not surprising that their deregulation might be associated to numerous disorders, e.g., inflammations, cancer, and NDDs. The starting point for CK1 inhibitors could be described as poorly selective and weakly potent molecules necessary to be improved for application in pharmacological treatment. Subsequently, compounds were developed, which show significant preference between the functionally different CK1 isoforms. Noteworthy, Pfizer designed two ATP-competitive compounds (PF-4800567 and PF-0670462), which possess selectivity towards the CK1δ and CK1ε isoforms. TG Therapeutics discovered umbralisib (UKONIQ™), an orally available dual inhibitor for PI3Kδ and CK1ε applied in the treatment of adults with relapsed or refractory marginal zone lymphoma, which received its FDA approval in 2021 (Burris et al., 2018). Despite this, there are no CK1 inhibitors reaching the clinical stage in neurodegenerative disorders. Those first successes raise the hope for the design of more selective and potent inhibitors of CK1 isoforms to improve the therapeutic opportunities.

In the case of TTBK1/2, up to date, only a small amount of molecules are known, which show potent inhibitory activity towards TTBK1/2. The undisputable advantage of TTBK1 over other kinases is its specific expression in neurons, and therefore, it seems to be a favorable target for NDDs.

Many kinds of CK2 inhibitors have been reported by using different methods, e.g., computer-aided drug design or structure-based reconstitution. Most of them lack cell permeability, high selectivity, but possess off-target potential. The latter might be explained by the fact that this kinase phosphorylates a huge amount of protein substrates. The principle characteristics for a satisfactory molecule are, furthermore, metabolic stability and a good pharmacokinetic profile. Even the best compound CX-4945, already in clinical use, is not devoid of unspecific effects. Nevertheless, the number of newly developed inhibitors (GO289, SGC-CK2-1, and the SRPIN803-rev derivatives), may increase the chance of developing highly selective and CNS penetrating molecules for CK2 in the near future.

## References

[B1] Ackermann K. Neidhart T. Gerber J. Waxmann A. Pyerin W. (2005). The catalytic subunit α′ gene of human protein kinase CK2 (CSNK2A2): Genomic organization, promoter identification and determination of Ets1 as a key regulator. Mol. Cell. Biochem. 274, 91–101. 10.1007/s11010-005-3076-2 16335532

[B2] Adler P. Mayne J. Walker K. Ning Z. Figeys D. (2019). Therapeutic targeting of casein kinase 1δ/ε in an Alzheimer’s disease mouse model. J. Proteome Res. 18, 3383–3393. 10.1021/acs.jproteome.9b00312 31334659

[B3] Ahmed T. Javed S. Javed S. Tariq A. Šamec D. Tejada S. (2017). Resveratrol and Alzheimer’s disease: Mechanistic insights. Mol. Neurobiol. 54, 2622–2635. 10.1007/s12035-016-9839-9 26993301

[B4] Akter R. Rahman H. Behl T. Chowdhury M. A. R. Manirujjaman M. Bulbul I. J. (2021). Prospective role of polyphenolic compounds in the treatment of neurodegenerative diseases. CNS Neurol. Disord. Drug Targets 20, 430–450. 10.2174/1871527320666210218084444 33602109

[B5] Alquezar C. Salado I. G. de la Encarnación A. Pérez D. I. Moreno F. Gil C. (2016). Targeting TDP-43 phosphorylation by casein kinase-1δ inhibitors: A novel strategy for the treatment of frontotemporal dementia. Mol. Neurodegener. 11, 36. 10.1186/s13024-016-0102-7 27138926 PMC4852436

[B6] Andrew S. E. Goldberg Y. P. Kremer B. Telenius H. Theilmann J. Adam S. (1993). The relationship between trinucleotide (CAG) repeat length and clinical features of Huntington’s disease. Nat. Genet. 4, 398–403. 10.1038/ng0893-398 8401589

[B7] Arai T. Hasegawa M. Akiyama H. Ikeda K. Nonaka T. Mori H. (2006). TDP-43 is a component of ubiquitin-positive tau-negative inclusions in frontotemporal lobar degeneration and amyotrophic lateral sclerosis. Biochem. Biophys. Res. Commun. 351, 602–611. 10.1016/j.bbrc.2006.10.093 17084815

[B8] Ardito F. Giuliani M. Perrone D. Troiano G. Muzio L. L. (2017). The crucial role of protein phosphorylation in cell signaling and its use as targeted therapy (Review). Int. J. Mol. Med. 40, 271–280. 10.3892/ijmm.2017.3036 28656226 PMC5500920

[B9] Arenas J. Campos Y. Ribacoba R. Martín M. A. Rubio J. C. Ablanedo P. (1998). Complex I defect in muscle from patients with Huntington’s disease. Ann. Neurol. 43, 397–400. 10.1002/ana.410430321 9506560

[B10] Atwal R. S. Desmond C. R. Caron N. Maiuri T. Xia J. Sipione S. (2011). Kinase inhibitors modulate huntingtin cell localization and toxicity. Nat. Chem. Biol. 7, 453–460. 10.1038/nchembio.582 21623356

[B11] Augustinack J. C. Schneider A. Mandelkow E. M. Hyman B. T. (2002). Specific tau phosphorylation sites correlate with severity of neuronal cytopathology in Alzheimer’s disease. Acta Neuropathol. 103, 26–35. 10.1007/s004010100423 11837744

[B12] Avraham E. Rott R. Liani E. Szargel R. Engelender S. (2007). Phosphorylation of parkin by the cyclin-dependent kinase 5 at the linker region modulates its ubiquitin-ligase activity and aggregation. J. Biol. Chem. 282, 12842–12850. 10.1074/jbc.M608243200 17327227

[B13] Ayala Y. M. Zago P. D’Ambrogio A. Xu Y.-F. Petrucelli L. Buratti E. (2008). Structural determinants of the cellular localization and shuttling of TDP-43. J. Cell Sci. 121, 3778–3785. 10.1242/jcs.038950 18957508

[B14] Ayala-Aguilera C. C. Valero T. lvaro Lorente-Macías Á. Baillache D. J. Croke S. Unciti-Broceta A. (2022). Small molecule kinase inhibitor drugs (1995–2021): Medical indication, pharmacology, and synthesis. J. Med. Chem. 65, 1047–1131. 10.1021/acs.jmedchem.1c00963 34624192

[B15] Aziz N. A. van der Burg J. M. M. Landwehrmeyer G. B. Brundin P. Stijnen T. EHDI Study Group (2008). Weight loss in Huntington disease increases with higher CAG repeat number. Neurology 71, 1506–1513. 10.1212/01.wnl.0000334276.09729.0e 18981372

[B16] Badura L. Swanson T. Adamowicz W. Adams J. Cianfrogna J. Fisher K. (2007). An inhibitor of casein kinase I induces phase delays in circadian rhythms under free-running and entrained conditions. J. Pharmacol. Exp. Ther. 322, 730–738. 10.1124/jpet.107.122846 17502429

[B17] Baier A. Galicka A. Nazaruk J. Szyszka R. (2017). Selected flavonoid compounds as promising inhibitors of protein kinase CK2α and CK2α’, the catalytic subunits of CK2. Phytochemistry 136, 39–45. 10.1016/j.phytochem.2016.12.018 28043654

[B18] Baier A. Nazaruk J. Galicka A. Szyszka R. (2018). Inhibitory influence of natural flavonoids on human protein kinase CK2 isoforms: Effect of the regulatory subunit. Mol. Cell. Biochem. 444, 35–42. 10.1007/s11010-017-3228-1 29188536 PMC6002439

[B19] Baier A. Szyszka R. (2020). Compounds from natural sources as protein kinase inhibitors. Biomolecules 10 (11), 1546. 10.3390/biom10111546 PMC7698043 33198400

[B20] Bailly C. (2015). Anticancer properties of lamellarins. Mar. Drugs 13, 1105–1123. 10.3390/md13031105 25706633 PMC4377975

[B21] Bain J. Plater L. Elliott M. Shpiro N. Hastie C. J. McLauchlan H. (2007). The selectivity of protein kinase inhibitors: A further update. Biochem. J. 408, 297–315. 10.1042/BJ20070797 17850214 PMC2267365

[B22] Ball B. J. Stein A. S. Borthakur G. Murray C. Kook K. Chan K. W. H. (2020). Trial in progress: A phase I trial of BTX-A51 in patients with relapsed or refractory aml or high-risk mds. Blood 136, 18–19. 10.1182/blood-2020-142557

[B23] Baluchnejadmojarad T. Rabiee N. Zabihnejad S. Roghani M. (2017). Ellagic acid exerts protective effect in intrastriatal 6-hydroxydopamine rat model of Parkinson’s disease: Possible involvement of ERβ/Nrf2/HO-1 signaling. Brain Res. 1662, 23–30. 10.1016/j.brainres.2017.02.021 28238669

[B24] Bao C. Bajrami B. Marcotte D. J. Chodaparambil J. V. Kerns H. M. Henderson J. (2021). Mechanisms of regulation and diverse activities of tau-tubulin kinase (TTBK) isoforms. Cell. Mol. Neurobiol. 41, 669–685. 10.1007/s10571-020-00875-6 32424773 PMC11448672

[B25] Barmada S. J. Skibinski G. Korb E. Rao E. J. Wu J. Y. Finkbeiner S. (2010). Cytoplasmic mislocalization of TDP-43 is toxic to neurons and enhanced by a mutation associated with familial amyotrophic lateral sclerosis. J. Neurosci. 30, 639–649. 10.1523/jneurosci.4988-09.2010 20071528 PMC2821110

[B26] Baunbæk D. Trinkler N. Ferandin Y. Lozach O. Ploypradith P. Rucirawat S. (2008). Anticancer alkaloid lamellarins inhibit protein kinases. Mar. Drugs 6, 514–527. 10.3390/md20080026 19172192 PMC2630805

[B27] Benn C. L. Dawson L. A. (2020). Clinically precedented protein kinases: Rationale for their use in neurodegenerative disease. Front. Aging Neurosci. 12, 242. 10.3389/fnagi.2020.00242 33117143 PMC7494159

[B28] Bibian M. Rahaim R. J. Choi J. Y. Noguchi Y. Schürer S. Chen W. (2013). Development of highly selective casein kinase 1δ/1ε (CK1δ/ε) inhibitors with potent antiproliferative properties. Bioorg. Med. Chem. Lett. 23, 4374–4380. 10.1016/j.bmcl.2013.05.075 23787102 PMC3783656

[B29] Bichelmeier U. Schmidt T. Hübener J. Boy J. Rüttiger L. Häbig K. (2007). Nuclear localization of ataxin-3 is required for the manifestation of symptoms in SCA3: *In vivo* evidence. J. Neurosci. 27, 7418–7428. 10.1523/JNEUROSCI.4540-06.2007 17626202 PMC6672614

[B30] Binder L. I. Frankfurter A. Rebhun L. I. (1985). The distribution of tau in the mammalian central nervous system. J. Cell Biol. 101, 1371–1378. 10.1083/jcb.101.4.1371 3930508 PMC2113928

[B31] Bingham E. W. Farrel H. M. Jr. (1974). Casein kinase from the Golgi apparatus of lactating mam mary gland. J. Biol. Chem. 249, 3647–3651. 10.1016/S0021-9258(19)42622-7 4364664

[B32] Bischof J. Leban J. Zaja M. Grothey A. Radunsky B. Othersen O. (2012). 2-Benzamido-*N*-(1*H*-benzo[*d*]imidazol-2-yl)thiazole-4-carboxamide derivatives as potent ihnhibitors CK1δ/ε. Amino Acids 43, 1577–1591. 10.1007/s00726-012-1234-x 22331384 PMC3448056

[B33] Blanquet P. R. (2000). Casein kinase 2 as a potentially important enzyme in the nervous system. Prog. Neurobiol. 60, 211–246. 10.1016/s0301-0082(99)00026-x 10658642

[B34] Borgo C. Cesaro L. Hirota T. Kuwata K. D’Amore C. Ruppert T. (2021a). Comparing the efficacy and selectivity of CK2 inhibitors. A phosphoproteomics approach. Eur. J. Med. Chem. 214, 113217. 10.1016/j.ejmech.2021.113217 33548633

[B35] Borgo C. D’Amore C. Sarno S. Salvi M. Ruzzene M. (2021b). Protein kinase CK2: A potential therapeutic target for diverse human diseases. Signal Transduct. Target. Ther. 6, 183. 10.1038/s41392-021-00567-7 33994545 PMC8126563

[B37] Bouskila M. Esoof N. Gay L. Fang E. H. Deak M. Begley M. J. (2011). TTBK2 kinase substrate specificity and the impact of spinocerebellar-ataxia-causing mutations on expression, activity, localization and development. Biochem. J. 437, 157–167. 10.1042/bj20110276 21548880 PMC3739326

[B38] Bowie E. Goetz S. C. (2020). TTBK2 and primary cilia are essential for the connectivity and survival of cerebellar Purkinje neurons. Elife 9, e51166. 10.7554/eLife.51166 31934864 PMC7028366

[B39] Boyarko B. Hook V. (2021). Human tau isoforms and proteolysis for production of toxic tau fragments in neurodegeneration. Front. Neurosci. 15, 702788. 10.3389/fnins.2021.702788 34744602 PMC8566764

[B40] Braak H. Braak E. (1991). Neuropathological stageing of Alzheimer-related changes. Acta Neuropathol. 82, 239–259. 10.1007/BF00308809 1759558

[B41] Braggin J. E. Bucks S. A. Course M. M. Smith C. L. Sopher B. Osnis L. (2019). Alternative splicing in a presenilin 2 variant associated with Alzheimer disease. Ann. Clin. Transl. Neurol. 6, 762–777. 10.1002/acn3.755 31020001 PMC6469258

[B42] Buontempo F. Orsini E. Lonetti A. Capellini A. Chiarini F. Evangelisti C. (2016). Synergistic cytotoxic effects of bortezomib and CK2 inhibitor CX-4945 in acute lymphoblastic leukemia: Turning off the prosurvival ER chaperone BIP/Grp78 and turning on the pro-apoptotic NF-κB. Oncotarget 7, 1323–1340. 10.18632/oncotarget.6361 26593250 PMC4811463

[B43] Burnett B. Li F. Pittman R. N. (2003). The polyglutamine neurodegenerative protein ataxin-3 binds polyubiquitylated proteins and has ubiquitin protease activity. Hum. Mol. Genet. 12, 3195–3205. 10.1093/hmg/ddg344 14559776

[B44] Burnett G. Kennedy E. P. (1954). The enzymatic phosphorylation of proteins. J. Biol. Chem. 211, 969–980. 10.1016/S0021-9258(18)71184-8 13221602

[B45] Burré J. Sharma M. Südhof T. C. (2018). Cell biology and pathophysiology of α-synuclein. Cold Spring Harb. Perspect. Med. 8, a024091. 10.1101/cshperspect.a024091 28108534 PMC5519445

[B46] Burris H. A. Flinn I. W. Patel M. R. Fenske T. S. Deng C. Brander D. M. (2018). Umbralisib, a novel PI3Kδ and casein kinase-1ε inhibitor, in relapsed or refractory chronic lymphocytic leukaemia and lymphoma: An open-label, phase 1, dose-escalation, first-in-human study. Lancet. Oncol. 19, 486–496. 10.1016/s1470-2045(18)30082-2 29475723

[B47] Burzio V. Antonelli M. Allende C. C. Allende J. E. (2002). Biochemical and cellular characteristics of the four splice variants of protein kinase CK1alpha from zebrafish (*Danio rerio*). J. Cell. Biochem. 86, 805–814. 10.1002/jcb.10263 12210746

[B48] Caltagirone C. Cisari C. Schievano C. Di Paola R. Cordaro M. Bruschetta G. (2015). Co-Ultramicronized palmitoylethanolamide/luteolin in the treatment of cerebral ischemia: From rodent to man. Transl. Stroke Res. 7, 54–69. 10.1007/s12975-015-0440-8 26706245 PMC4720704

[B49] Carter B. Justin H. S. Gulick D. Gamsby J. J. (2021). The molecular clock and neurodegenerative disease: A stressful time. Front. Mol. Biosci. 8, 644747. 10.3389/fmolb.2021.644747 33889597 PMC8056266

[B50] Castello J. Ragnauth A. Friedman E. Rebholz H. (2017). CK2—an emerging target for neurological and psychiatric disorders. Pharmaceuticals 10, 7. 10.3390/ph10010007 PMC5374411 28067771

[B51] Chakraborty J. Basso V. Ziviani E. (2017). Post translational modification of Parkin. Biol. Direct 12, 6. 10.1186/s13062-017-0176-3 28222786 PMC5319146

[B52] Chen C. Gu J. Basurto-Islas G. Jin N. Wu F. Gong C.-X. (2017). Up-regulation of casein kinase 1ε is involved in tau pathogenesis in Alzheimer’s disease. Sci. Rep. 7, 13478. 10.1038/s41598-017-13791-5 29044200 PMC5647372

[B53] Chen G.-F. Xu T.-H. Yan Y. Zhou Y.-R. Jiang Y. Melcher K. (2017). Amyloid beta: Structure, biology and structure-based therapeutic development. Acta Pharmacol. Sin. 38, 1205–1235. 10.1038/aps.2017.28 28713158 PMC5589967

[B54] Cheong J. K. Hung N. T. Wang H. Tan P. Voorhoeve P. M. Lee S. H. (2011). IC261 induces cell cycle arrest and apoptosis of human cancer cells via CK1δ/ɛ and Wnt/β-catenin independent inhibition of mitotic spindle formation. Oncogene 30, 2558–2569. 10.1038/onc.2010.627 21258417 PMC3109269

[B55] Chohan M. O. Khatoon S. Iqbal I.-G. Iqbal K. (2006). Involvement of I2PP2A in the abnormal hyperphosphorylation of tau and its reversal by Memantine. FEBS Lett. 580, 3973–3979. 10.1016/j.febslet.2006.06.021 16806196

[B56] Chon H. J. Bae K. J. Lee Y. Kim J. (2015). The casein kinase 2 inhibitor, CX-4945, as an anti-cancer drug in treatment of human hematological malignancies. Front. Pharmacol. 6, 70. 10.3389/fphar.2015.00070 25873900 PMC4379896

[B57] Citron M. Diehl T. S. Gordon G. Biere A. L. Seubert P. Selkoe D. J. (1996). Evidence that the 42- and 40-amino acid forms of amyloid β protein are generated from the β-amyloid precursor protein by different protease activities. Proc. Natl. Acad. Sci. U. S. A. 93, 13170–13175. 10.1073/pnas.93.23.13170 8917563 PMC24065

[B58] Cohen P. (2002). The origins of protein phosphorylation. Nat. Cell Biol. 4, E127–E130. 10.1038/ncb0502-e127 11988757

[B59] Conway K. A. Harper J. D. Lansbury P. T. (1998). Accelerated *in vitro* fibril formation by a mutant alpha-synuclein linked to early-onset Parkinson disease. Nat. Med. 4, 1318–1320. 10.1038/3311 9809558

[B60] Cookson M. R. Lockhart P. J. McLendon C. O’Farrell C. Schlossmacher M. Farrer M. J. (2003). RING finger 1 mutations in parkin produce altered localization of the protein. Hum. Mol. Genet. 12, 2957–2965. 10.1093/hmg/ddg328 14519684

[B61] Cornett J. Cao F. Wang C. E. Ross C. A. Bates G. P. Li S. H. (2005). Polyglutamine expansion of huntingtin impairs its nuclear export. Nat. Genet. 37, 198–204. 10.1038/ng1503 15654337

[B62] Costa P. S. D. Ramos P. S. Fereira C. Silva J. L. El-Baha T. Fialho E. (2021). Pro-oxidant efeect of resveratrol on human breast cancer MCF-7 cells is associated with CK2 inhibition. Nutr. Cancer 14, 1–10. 10.1080/016355581.2021.1977834 34519606

[B63] Cozza G. Bonvini P. Zorzi E. Poletto G. Pagano M. A. Sarno S. (2006). Identification of ellagic acid as potent inhibitor of protein kinase CK2: A successful example of a virtual screening application. J. Med. Chem. 49, 2363–2366. 10.1021/jm060112m 16610779

[B64] Cozza G. Gianoncelli A. Montopoli M. Caparrotta L. Venerando A. Meggio F. (2008). Identification of novel protein kinase CK1 delta (CK1delta) inhibitors through structure-based virtual screening. Bioorg. Med. Chem. Lett. 18, 5672–5675. 10.1016/j.bmcl.2008.08.072 18799313

[B65] Cozza G. Mazzorana M. Papinutto E. Bain J. Elliott M. di Maira G. (2009). Quinalizarin as a potent, selective and cell-permeable inhibitor of protein kinase CK2. Biochem. J. 421, 387–395. 10.1042/bj20090069 19432557

[B66] Cozza G. Pinna L. A. (2016). Casein kinases as potential therapeutic targets. Expert Opin. Ther. Targets 20, 319–340. 10.1517/14728222.2016.1091883 26565594

[B67] Cozza G. Pinna L. A. Moro S. (2012). Protein kinase CK2 inhibitors: A patent review. Expert Opin. Ther. Pat. 22, 1081–1097. 10.1517/13543776.2012.717615 22908959

[B68] Cozza G. Venerando A. Sarno S. Pinna L. A. (2015). The selectivity of CK2 inhibitor quinalizarin: A reevaluation. Biomed. Res. Int. 2015, 734127. 10.1155/2015/734127 26558278 PMC4628998

[B69] Cunningham P. S. Ahern S. A. Smith L. C. da Silva Santos C. S. Wager T. T. Bechtold D. A. (2016). Targeting of the circadian clock via CK1δ/ε to improve glucose homeostasis in obesity. Sci. Rep. 6, 29983. 10.1038/srep29983 27439882 PMC4954991

[B70] Daily J. W. Kang S. Park S. (2021). Protection against Alzheimer's disease by luteolin: Role of brain glucose regulation, anti-inflammatory activity, and the gut microbiota-liver-brain axis. Biofactors 47, 218–231. 10.1002/biof.1703 33347668

[B71] Dalle Vedove A. Zonta F. Zanforlin E. Demitri N. Ribaudo G. Cazzanelli G. (2020). A novel class of selective CK2 inhibitors targeting its open hinge conformation. Eur. J. Med. Chem. 195, 112267. 10.1016/j.ejmech.2020.112267 32283296

[B72] De Wit T. Baekelandt V. Lobbestael E. (2018). Inhibition of LRRK2 or casein kinase 1 results in LRRK2 protein destabilization. Mol. Neurobiol. 56, 5273–5286. 10.1007/s12035-018-1449-2 30592011 PMC6657425

[B73] Desmond C. R. Atwal R. S. Xia J. Truant R. (2012). Identification of a karyopherin β1/β2 proline-tyrosine nuclear localization signal in huntingtin protein. J. Biol. Chem. 287, 39626–39633. 10.1074/jbc.M112.412379 23012356 PMC3501053

[B74] Dheen S. T. Jun Y. Yan Z. Tay S. S. W. Ang Ling E. (2005). Retinoic acid inhibits expression of TNF-α and iNOS in activated rat microglia. Glia 50, 21–31. 10.1002/glia.20153 15602748

[B75] Dillon G. M. Henderson J. L. Bao C. Joyce J. A. Calhoun M. Amaral B. (2020). Acute inhibition of the CNS-specific kinase TTBK1 significantly lowers tau phosphorylation at several disease relevant sites. PLOS ONE 15 (4), e0228771. 10.1371/journal.pone.0228771 32255788 PMC7138307

[B76] Domańska K. Zieliński R. Kubiński K. Sajnaga E. Masłyk M. Bretner M. (2005). Different properties of four molecular forms of protein kinase CK2 from *Saccharomyces cerevisiae* . Acta Biochim. Pol. 52, 947–952. 10.18388/abp.2005_3413 16265593

[B77] Duan G. Walther D. (2015). The roles of post-translational modifications in the context of protein interaction networks. PLoS Comput. Biol. 11, e1004049. 10.1371/journal.pcbi.1004049 25692714 PMC4333291

[B78] Ebrahimi R. Sepand M. R. Seyednejad S. A. Omidi A. Akbariani M. Gholami M. (2019). Ellagic acid reduces methotrexate-induced apoptosis and mitochondrial dysfunction via up-regulating Nrf2 expression and inhibiting the IĸBα/NFĸB in rats. DARU 27, 721–733. 10.1007/s40199-019-00309-9 31736017 PMC6895372

[B79] El-Agnaf O. M. Jakes R. Curran M. D. Wallace A. (1998). Effects of the mutations Ala30 to Pro and Ala53 to Thr on the physical and morphological properties of α-synuclein protein implicated in Parkinson’s disease. FEBS Lett. 440, 67–70. 10.1016/s0014-5793(98)01419-7 9862427

[B80] Ellis C. E. Schwartzberg P. L. Grider T. L. Fink D. W. Nussbaum R. L. (2001). alpha-synuclein is phosphorylated by members of the Src family of protein-tyrosine kinases. J. Biol. Chem. 276, 3879–3884. 10.1074/jbc.M010316200 11078745

[B81] Evert B. O. Araujo J. Vieira-Saecker A. M. de Vos R. A. Harendza S. Klockgether T. (2006). Ataxin-3 represses transcription via chromatin binding, interaction with histone deacetylase 3, and histone deacetylation. J. Neurosci. 26, 11474–11486. 10.1523/JNEUROSCI.2053-06.2006 17079677 PMC6674535

[B82] Evin G. Smith M. J. Tziotis A. McLean C. Canterford L. Sharples R. A. (2002). Alternative transcripts of presenilin-1 associated with frontotemporal dementia. Neuroreport 13, 917–921. 10.1097/00001756-200205070-00036 11997713

[B83] Farkhondeh T. Folgado S. L. Pourbagher-Shahri A. M. Ashrafizadeh M. Samarghandian S. (2020). The therapeutic effect of resveratrol: Focusing on the Nrf2 signaling pathway. Biomed. Pharmacother. 127, 110234. 10.1016/j.biopha.2020.110234 32559855

[B84] Flajolet M. He G. Heiman M. Lin A. Nairn A. C. Greengard P. (2007). Regulation of Alzheimer’s disease amyloid-beta formation by casein kinase I. Proc. Natl. Acad. Sci. U. S. A. 104, 4159–4164. 10.1073/pnas.0611236104 17360493 PMC1820725

[B85] Flotow H. Roach P. J. (1991). Role of acidic residues as substrate determinants for casein kinase I. J. Biol. Chem. 266, 3724–3727. 10.1016/S0021-9258(19)67854-3 1995625

[B86] Franchin C. Cesaro L. Salvi M. Millioni R. Iori E. Cifani P. (2015). Quantitative analysis of a phosphoproteome readily altered by the protein kinase CK2 inhibitor quinalizarin in HEK-293T cells. Biochim. Biophys. Acta 1854, 609–623. 10.1016/j.bbapap.2014.09.017 25278378

[B87] Fujiwara H. Hasegawa M. Dohmae N. Kawashima A. Masliah E. Goldberg M. S. (2002). Alpha-Synuclein is phosphorylated in synucleinopathy lesions. Nat. Cell Biol. 4, 160–164. 10.1038/ncb748 11813001

[B88] Fukuda T. Ishibashi F. Iwao M. (2020). Lamellarin alkaloids: Isolation, synthesis, and biological activity. Alkaloids. Chem. Biol. 83, 1–112. 10.1016/bs.alkal.2019.10.001 32098648

[B89] Fulcher L. J. Sapkota G. P. (2020). Functions and regulation of the serine/threonine protein kinase CK1 family: Moving beyond promiscuity. Biochem. J. 477, 4603–4621. 10.1042/BCJ20200506 33306089 PMC7733671

[B90] Gai W. P. Power J. H. Blumbergs P. C. Blessing W. W. (1998). Multiple-system atrophy: A new a-synuclein disease? Lancet 352, 547–548. 10.1016/s0140-6736(05)79256-4 9716068

[B91] García-Reyes B. Witt L. Jansen B. Karasu E. Gehring T. Leban J. (2018). Discovery of inhibitor of Wnt production 2 (IWP-2) and related compounds as selective ATP-competitive inhibitors of casein kinase 1 (CK1) δ/ε. J. Med. Chem. 61, 4087–4102. 10.1021/acs.jmedchem.8b00095 29630366

[B92] Geuens T. Bouhy D. Timmerman V. (2016). The hnRNP family: Insights into their role in health and disease. Hum. Genet. 135, 851–867. 10.1007/s00439-016-1683-5 27215579 PMC4947485

[B93] Ghoshal N. Smiley J. F. DeMaggio A. J. Hoekstra M. F. Cochran E. J. Binder L. I. (1999). A new molecular link between the fibrillar and granulovacuolar lesions of Alzheimer’s disease. Am. J. Pathol. 155, 1163–1172. 10.1016/s0002-9440(10)65219-4 10514399 PMC1867028

[B94] Glenner G. G. Wong C. W. (1984). Alzheimer’s disease: Initial report of the purification and characterization of a novel cerebrovascular amyloid protein. Biochem. Biophys. Res. Commun. 120, 885–890. 10.1016/s0006-291x(84)80190-4 6375662

[B95] Golub A. G. Bdzhola V. G. Briukhovetska N. V. Balanda A. O. Kukharenko O. P. Kotey I. M. (2011). Synthesis and biological evaluation of substituted (thieno[2, 3-d]pyrimidin-4-ylthio)carboxylic acids as inhibitors of human protein kinase CK2. Eur. J. Med. Chem. 46, 870–876. 10.1016/j.ejmech.2010.12.025 21276643

[B96] Gomes B. A. Q. Silva J. P. B. Romeiro C. F. R. dos Santos S. M. Rodrigues C. A. Gonçalves P. R. (2018). Neuroprotective mechanisms of resveratrol in Alzheimer’s disease: Role of SIRT1. Oxid. Med. Cell. Longev. 2018, 8152373. 10.1155/2018/8152373 30510627 PMC6232815

[B97] Gong C.-X. Iqbal K. (2008). Hyperphosphorylation of microtubule-associated protein tau: A promising therapeutic target for alzheimer disease. Curr. Med. Chem. 15, 2321–2328. 10.2174/092986708785909111 18855662 PMC2656563

[B98] Gong C.-X. Singh T. J. Grundke-Iqbal I. Iqbal K. (1993). Phosphoprotein phosphatase activities in Alzheimer disease brain. J. Neurochem. 61, 921–927. 10.1111/j.1471-4159.1993.tb03603.x 8395566

[B99] Greenwood J. A. Scott C. W. Spreen R. C. Caputo C. B. Johnson G. V. (1994). Casein kinase II preferentially phosphorylates human tau isoforms containing an amino-terminal insert. Identification of threonine 39 as the primary phosphate acceptor. J. Biol. Chem. 269, 4373–4380. 10.1016/S0021-9258(17)41790-x 8308007

[B100] Grundke-Iqbal I. Iqbal K. Tung Y. C. Quinlan M. Wisniewski H. M. Binder L. I. (1986). Abnormal phosphorylation of the microtubule-associated protein tau (tau) in Alzheimer cytoskeletal pathology. Proc. Natl. Acad. Sci. U. S. A. 83, 4913–4917. 10.1073/pnas.83.13.4913 3088567 PMC323854

[B101] Guo D.-J. Li F. Yu P. H.-F. Chan S.-W. (2013). Neuroprotective effects of luteolin against apoptosis induced by 6-hydroxydopamine on rat pheochromocytoma PC12 cells. Pharm. Biol. 51, 190–196. 10.3109/13880209.2012.716852 23035972

[B102] Hackam A. S. Singaraja R. Wellington C. L. Metzler M. McCutcheon K. Zhang T. (1998). The influence of huntingtin protein size on nuclear localization and cellular toxicity. J. Cell Biol. 141, 1097–1105. 10.1083/jcb.141.5.1097 9606203 PMC2137174

[B103] Haidar S. Aichele D. Birus R. Hielscher J. Laitinen T. Poso A. (2019). *In vitro* and *in silico* evaluation of bikaverin as a potent inhibitor of human protein kinase CK2. Molecules 24, 1380. 10.3390/molecules24071380 PMC6479664 30965682

[B104] Halekotte J. Witt L. Ianes C. Krüger M. Bührmann M. Rauh D. (2017). Optimized 4, 5-diarylimidazoles as potent/selective inhibitors of protein kinase CK1δ and their structural relation to p38α MAPK. Molecules 22, 522. 10.3390/molecules22040522 PMC6154583 28338621

[B105] Halkina T. Henderson J. L. Lin E. Y. Himmelbauer M. K. Jones J. H. Nevalainen M. (2021). Discovery of potent and brain-penetrant tau tubulin kinase 1 (TTBK1) inhibitors that lower tau phosphorylation *in vivo* . J. Med. Chem. 64, 6358–6380. 10.1021/acs.jmedchem.1c00382 33944571

[B106] Hampe C. Ardila-Osorio H. Fournier M. Brice A. Corti O. (2006). Biochemical analysis of Parkinson’s disease-causing variants of parkin, an E3 ubiquitin-protein ligase with monoubiquitylation capacity. Hum. Mol. Genet. 15, 2059–2075. 10.1093/hmg/ddl131 16714300

[B107] Hanger D. P. Anderton B. H. Noble W. (2009). Tau phosphorylation: The therapeutic challenge for neurodegenerative disease. Trends Mol. Med. 15, 112–119. 10.1016/j.molmed.2009.01.003 19246243

[B108] Hanger D. P. Byers H. L. Wray S. Leung K.-Y. Saxton M. J. Seereeram A. (2007). Novel phosphorylation sites in tau from alzheimer brain support a role for casein kinase 1 in disease pathogenesis. J. Biol. Chem. 282, 23645–23654. 10.1074/jbcM703269200 17562708

[B109] Hanks S. K. Hunter T. (1995). The eukaryotic protein kinase superfamily: Kinase (catalytic) domain structure and classification ^1^ . FASEB J. 9, 576–596. 10.1096/fasebj.9.8.7768349 7768349

[B110] Hardy J. A. Higgins G. A. (1992). Alzheimer’s disease: The amyloid cascade hypothesis. Science 1256, 184–185. 10.1126/science.1566067 1566067

[B111] Hasegawa M. Arai T. Nonaka T. Kametani F. Yoshida M. Hashizume Y. (2008). Phosphorylated TDP-43 in frontotemporal lobar degeneration and amyotrophic lateral sclerosis. Ann. Neurol. 64, 60–70. 10.1002/Ana.21425 18546284 PMC2674108

[B112] Hathaway G. M. Traugh J. A. (1979). Cyclic nucleotide-independent protein kinases from rabbit reticulocytes. Purification of casein kinases. J. Biol. Chem. 254, 762–768. 10.1016/S0021-9258(17)37871-7 216682

[B113] Hinojosa-Ventura G. Puebla-Pérez A. M. Gallegos-Arreola M. P. Chávez-Parga M.-C. Romero-Estrada A. Delgado-Saucedo J. I. (2019). Cytotoxic and antitumoral effects of bikaverin from Gibberella fujikuroi on L5178Y lymphoma murine model. J. Mex. Chem. Soc. 63, 115–122. 10.29356/jmcs.v63i4.729

[B114] Houlden H. Johnson J. Gardner-Thorpe C. Lashley T. Hernandez D. Worth P. (2007). Mutations in TTBK2, encoding a kinase implicated in tau phosphorylation, segregate with spinocerebellar ataxia type 11. Nat. Genet. 39, 1434–1436. 10.1038/ng.2007.43 18037885

[B115] Hua Z. Huang X. Bregman H. Chakka N. DiMauro E. F. Doherty E. M. (2012). 2-Phenylamino-6-cyano-1H-benzimidazole-based isoform selective casein kinase 1 gamma (CK1γ) inhibitors. Bioorg. Med. Chem. Lett. 22, 5392–5395. 10.1016/j.bmcl.2012.07.046 22877629

[B116] Hunter T. (1995). Protein kinases and phosphatases: The yin and yang of protein phosphorylation and signaling. Cell 80, 225–236. 10.1016/0092-8674(95)90405-0 7834742

[B117] Hunter T. (2012). Why nature chose phosphate to modify proteins. Philos. Trans. R. Soc. Lond. B Biol. Sci. 367, 2513–2516. 10.1098/rstb.2012.0013 22889903 PMC3415839

[B118] Ikezu S. Ikezu T. (2014). Tau-tubulin kinase. Front. Mol. Neurosci. 7, 33. 10.3389/fnmol.2014.00033 24808823 PMC4009424

[B119] Ikezu S. Ingraham Dixie K. L. Koro L. Watanabe T. Kaibuchi K. Ikezu T. (2020). Tau-tubulin kinase 1 and amyloid-β peptide induce phosphorylation of collapsin response mediator protein-2 and enhance neurite degeneration in Alzheimer disease mouse models. Acta Neuropathol. Commun. 8, 12. 10.1186/s40478-020-0890-4 32019603 PMC7001309

[B120] Inukai Y. Nonaka T. Arai T. Yoshida M. Hashizume Y. Beach T. G. (2008). Abnormal phosphorylation of ser409/410 of TDP-43 in FTLD-U and ALS. FEBS Lett. 582, 2899–2904. 10.1016/j.febslet.2008.07.027 18656473

[B121] Ishii A. Nonaka T. Taniguchi S. Saito T. Arai T. Mann D. (2007). Casein kinase 2 is the major enzyme in brain that phosphorylates Ser129 of human alpha-synuclein: Implication for alpha-synucleinopathies. FEBS Lett. 581, 4711–4717. 10.1016/j.febslet.2007.08.067 17868672

[B122] Iwao M. Fukuda T. Ishibashi F. (2011). Synthesis and biological activity of lamellarin alkaloids: An overview. HETEROCYCLES 83, 491. 10.3987/rev-10-686 32098648

[B123] Jackson P. K. (2012). TTBK2 kinase: Linking primary cilia and cerebellar ataxias. Cell 151, 697–699. 10.1016/j.cell.2012.10.027 23141531

[B124] Jameson L. Frey T. Zeeberg B. Dalldorf F. Caplow M. (1980). Inhibition of microtubule assembly by phosphorylation of microtubule-associated proteins. Biochemistry 19, 2472–2479. 10.1021/bi00552a027 7387985

[B125] Jana S. Singh S. K. (2020). Identification of human tau-tubulin kinase 1inhibitors: An integrated e-pharmacophore based virtual screening and molecular dynamics simulation. J. Biomol. Struct. Dyn. 38, 886–900. 10.1080/07391102.2019.1590242 30829560

[B126] Janeczko M. Orzeszko A. Kazimierczuk Z. Szyszka R. Baier A. (2012). CK2α and CK2α′ subunits differ in their sensitivity to 4, 5, 6, 7-tetrabromo- and 4, 5, 6, 7-tetraiodo-1H-benzimidazole derivatives. Eur. J. Med. Chem. 47, 345–350. 10.1016/j.ejmech.2011.11.002 22115617

[B127] Järås M. Miller P. G. Chu L. P. Puram R. V. Fink E. C. Schneider R. K. (2014). Csnk1a1 inhibition has p53-dependent therapeutic efficacy in acute myeloid leukemia. J. Exp. Med. 211, 605–612. 10.1084/jem.20131033 24616378 PMC3978274

[B128] Jiang S. Zhang M. Sun J. Yang X. (2018). Casein kinase 1α: Biological mechanisms and theranostic potential. Cell Commun. Signal. 16, 23. 10.1186/s12964-018-0236-z 29793495 PMC5968562

[B129] Jin J. Pawson T. (2012). Modular evolution of phosphorylation-based signalling systems. Philos. Trans. R. Soc. Lond. B Biol. Sci. 367, 2540–2555. 10.1098/rstb.2012.0106 22889906 PMC3415845

[B130] Kametani F. Nonaka T. Suzuki T. Arai T. Dohmae N. Akiyama H. (2009). Identification of casein kinase-1 phosphorylation sites on TDP-43. Biochem. Biophys. Res. Commun. 382, 405–409. 10.1016/j.bbrc.2009.03.038 19285963

[B131] Kang S. S. Lee J. Y. Choi Y. K. Kim G. S. Han B. H. (2004). Neuroprotective effects of flavones on hydrogen peroxide-induced apoptosis in SH-SY5Y neuroblostoma cells. Bioorg. Med. Chem. Lett. 14, 2261–2264. 10.1016/j.bmcl.2004.02.003 15081021

[B132] Kannanayakal T. J. Tao H. Vandre D. D. Kuret J. (2006). Casein kinase-1 isoforms differentially associate with neurofibrillary and granulovacuolar degeneration lesions. Acta Neuropathol. 111, 413–421. 10.1007/s00401-006-0049-9 16557393

[B133] Karve T. M. Cheema A. K. (2011). Small changes huge impact: The role of protein posttranslational modifications in cellular homeostasis and disease. J. Amino Acids 2011, 207691. 10.4061/2011/207691 22312457 PMC3268018

[B134] Kiefer S. E. Chang C. J. Kimura S. R. Gao M. Xie D. Zhang Y. (2014). The structure of human tau-tubulin kinase 1 both in the apo form and in complex with an inhibitor. Acta Crystallogr. F. Struct. Biol. Commun. 70, 173–181. 10.1107/S2053230X14000144 24637750 PMC3936456

[B135] Kim E. N. Lim J. H. Kim M. Y. Ban T. H. Jang I. Yoon H. E. (2018). Resveratrol, an Nrf2 activator, ameliorates aging-related progressive renal injury. Aging (Albany NY) 10, 83–99. 10.18632/aging.101361 29326403 PMC5811244

[B136] Kim H. Choi K. Kang H. Lee S.-Y. Chi S.-W. Lee M.-S. (2014). Identification of a novel function of CX-4945 as a splicing regulator. PLoS One 9, e94978. 10.1371/journal.pone.0094978 24743259 PMC3990583

[B137] Kim J. K. Park S. U. (2021). Flavonoids for treatment of Alzheimer’s disease: An up todate review. EXCLI J. 20, 495–502. 10.17179/excli2021-3492 33883978 PMC8056054

[B138] Knippschild U. Gocht A. Wolff S. Huber N. Löhler J. Stöter M. (2005). The casein kinase 1 family: Participation in multiple cellular processes in eukaryotes. Cell. Signal. 17, 675–689. 10.1016/j.cellsig.2004.12.011 15722192

[B139] Komorowska J. Wątroba M. Szukiewicz D. (2020). Review of beneficial effects of resveratrol in neurodegenerative diseases such as Alzheimer’s disease. Adv. Med. Sci. 65, 415–423. 10.1016/j.advms.2020.08.002 32871321

[B140] Kosten J. Binolfi A. Stuiver M. Verzini S. Theillet F.-X. Bekei B. (2014). Efficient modification of alpha-synuclein serine 129 by protein kinase CK1 requires phosphorylation of tyrosine 125 as a priming event. ACS Chem. Neurosci. 5, 1203–1208. 10.1021/cn5002254 25320964

[B141] Kotanidou A. Xagorari A. Bagli E. Kitsanta P. Fotsis T. Papapetropoulos A. (2002). Luteolin reduces lipopolysaccharide-induced lethal toxicity and expression of proinflammatory molecules in mice. Am. J. Respir. Crit. Care Med. 165, 818–823. 10.1164/ajrccm.165.6.2101049 11897650

[B142] Kovacs G. G. Botond G. Budka H. (2010). Protein coding of neurodegenerative dementias: The neuropathological basis of biomarker diagnostics. Acta Neuropathol. 119, 389–408. 10.1007/s00401-010-0658-1 20198481

[B326] Kumar A. Singh C. K. LaVoie H. A. DiPette D. J. Singh U. S. (2011). Resveratrol Restores Nrf2 Level and Prevents Ethanol-Induced Toxic Effects in the Cerebellum of a Rodent Model of Fetal Alcohol Spectrum Disorders. Mol. Pharmacol. 80, 446–457. 10.1124/mol.111.071126 21697273 PMC3164333

[B144] Kwon Y. (2017). Luteolin as a potential preventive and therapeutic candidate for Alzheimer’s disease. Exp. Gerontol. 95, 39–43. 10.1016/j.exger.2017.05.014 28528007

[B145] LaFerla F. M. Green K. N. Oddo S. (2007). Intracellular amyloid-β in Alzheimer’s disease. Nat. Rev. Neurosci. 8, 499–509. 10.1038/nrn2168 17551515

[B146] Lasa M. Marin O. Pinna L. A. (1997). Rat liver Golgi apparatus contains a protein kinase similar to the casein kinase of lactating mam mary gland. Eur. J. Biochem. 243, 719–725. 10.1111/j.1432-1033.1997.00719.x 9057837

[B147] Lastres-Becker I. García-Yagüe A. J. Scannevin R. H. Casarejos M. J. Kügler S. Rábano A. (2016). Repurposing the NRF2 activator dimethyl fumarate as therapy against synucleinopathy in Parkinson’s disease. Antioxid. Redox Signal. 25, 61–77. 10.1089/ars.2015.6549 27009601 PMC4943471

[B148] Ławnicka H. Kowalewicz-Kulbat M. Sicińska P. Kazimierczuk Z. Grieb P. Stępień P. (2010). Anti-neoplastic effect of protein kinase CK2 inhibitor, 2-dimethylamino-4, 5, 6, 7-tetrabromobenzimidazole (DMAT), on growth and hormonal activity of human adrenocortical carcinoma cell line (H295R) *in vitro* . Cell Tissue Res. 340, 371–379. 10.1007/s00441-010-0960-1 20383646

[B149] Lee J. W. Hirota T. Peters E. C. Garcia M. Gonzalez R. Cho C. Y. (2011). A small molecule modulates circadian rhythms through phosphorylation of the period protein. Angew. Chem. Int. Ed. Engl. 50, 10608–10611. 10.1002/anie.201103915 21954091 PMC3755734

[B150] Lee J. Y. Yun J.-S. Kim W.-K. Chun H.-S. Jin H. Cho S. (2019). Structural basis for the selective inhibition of cdc2-like kinases by CX-4945. Biomed. Res. Int. 2019, 6125068. 10.1155/2019/6125068 31531359 PMC6720368

[B151] Lee V. M. Goedert M. Trojanowski J. Q. (2001). Neurodegenerative tauopathies. Annu. Rev. Neurosci. 24, 1121–1159. 10.1146/annurev.neuro.24.1.1121 11520930

[B152] Li C. Liu X. Lin X. Chen X. (2009). Structure-activity relationship of 7 flavonoids on recombinant human protein kinase CK2 holoenzyme. Zhong Nan Da Xue Xue Bao Yi Xue Ban. 34, 20–26. 19197122

[B153] Li G. Yin H. Kurent J. (2004). Casein kinase 1 delta phosphorylates tau and disrupts its binding to microtubules. J. Biol. Chem. 279, 15938–15945. 10.1074/jbc.M314116200 14761950

[B154] Li H.-Y. Yeh P.-A. Chiu H.-C. Tang C.-Y. Tu B. P.-h. (2011). Hyperphosphorylation as a defense mechanism to reduce TDP-43 aggregation. PLoS One 6, e23075. 10.1371/journal.pone.0023075 21850253 PMC3151276

[B155] Li K. McGee L. R. Fisher B. Sudom A. Liu J. Rubenstein S. M. (2013). Inhibiting NF-κB-inducing kinase (NIK): Discovery, structure-based design, synthesis, structure–activity relationship, and co-crystal structures. Bioorg. Med. Chem. Lett. 23, 1238–1244. 10.1016/j.bmcl.2013.01.012 23374866

[B156] Li M. Makkinje A. Damuni Z. (1996). Molecular identification of I1PP2A, a novel potent heat-stable inhibitor protein of protein phosphatase 2A. Biochemistry 35, 6998–7002. 10.1021/bi960581y 8679524

[B157] Liachko N. F. McMillan P. J. Strovas T. J. Loomis E. Greenup L. Murrell J. R. (2014). The tau tubulin kinases TTBK1/2 promote accumulation of pathological TDP-43. PLoS Genet. 10, e1004803. 10.1371/journal.pgen.1004803 25473830 PMC4256087

[B158] Liao J.-C. Yang T. T. Weng R. R. Kuo C.-T. Chang C.-W. (2015). TTBK2: A tau protein kinase beyond tau phosphorylation. Biomed. Res. Int. 2015, 575170. 10.1155/2015/575170 25950000 PMC4407412

[B159] Lindwall G. Cole R. D. (1984). Phosphorylation affects the ability of tau protein to promote microtubule assembly. J. Biol. Chem. 259, 5301–5305. 10.1016/S0021-9258(17)42989-9 6425287

[B160] Lippa C. F. Fujiwara H. Mann D. M. Giasson B. Baba M. Schmidt M. L. (1998). Lewy bodies contain altered alpha-synuclein in brains of many familial Alzheimer's disease patients with mutations in presenilin and amyloid precursor protein genes. Am. J. Pathol. 153, 1365–1370. 10.1016/s0002-9440(10)65722-7 9811326 PMC1853391

[B161] Litchfield D. W. (2003). Protein kinase CK2: Structure, regulation and role in cellular decisions of life and death. Biochem. J. 369, 1–15. 10.1042/bj20021469 12396231 PMC1223072

[B162] Liu G. Li H. Zhang W. Yu J. Zhang X. Wu R. (2021). Csnk1a1 inhibition modulates the inflammatory secretome and enhances response to radiotherapy in glioma. J. Cell. Mol. Med. 25, 7395–7406. 10.1111/jcmm.16767 34216174 PMC8335695

[B163] Liu J. Yi L. Xiang Z. Zhong J. Zhang H. Sun T. (2015). Resveratrol attenuates spinal cord injury-induced inflammatory damage in rat lungs. Int. J. Clin. Exp. Pathol. 8, 1237–1246. 25973008 PMC4396266

[B164] Liu Q.-S. Deng R. Li S. Li X. Li K. Kebaituli G. (2017). Ellagic acid protects against neuron damage in ischemic stroke through regulating the ratio of Bcl-2/Bax expression. Appl. Physiol. Nutr. Metab. 42, 855–860. 10.1139/apnm-2016-0651 28388366

[B165] Lolli G. Cozza G. Mazzorana M. Tibaldi E. Cesaro L. Donella-Deana A. (2012). Inhibition of protein kinase CK2 by flavonoids and tyrphostins. A structural insight. Biochemistry 51, 6097–6107. 10.1021/bi300531c 22794353

[B166] Lopez-Lazaro M. (2009). Distribution and biological activities of the flavonoid luteolin. Mini Rev. Med. Chem. 9, 31–59. 10.2174/138955709787001712 19149659

[B167] Lund H. Cowburn R. F. Gustafsson E. Strömberg K. Svensson A. Dahllund L. (2013). Tau-tubulin kinase 1 expression, phosphorylation and Co-localization with phospho-ser422 tau in the Alzheimer’s disease brain. Brain Pathol. 23, 378–389. 10.1111/bpa.12001 23088643 PMC8029021

[B168] Lunkes A. Lindenberg K. S. Ben-Haїem L. Weber C. Devys D. Landwehrmeyer G. B. (2002). Proteases acting on mutant huntingtin generate cleaved products that differentially build up cytoplasmic and nuclear inclusions. Mol. Cell 10, 259–269. 10.1016/s1097-2765(02)00602-0 12191472

[B169] Mackenzie I. R. Rademakers R. Neumann M. (2010). TDP-43 and FUS in amyotrophic lateral sclerosis and frontotemporal dementia. Lancet. Neurol. 9, 995–1007. 10.1016/S1474-4422(10)70195-2 20864052

[B170] Maher P. Dargusch R. Bodai L. Gerard P. E. Purcell J. M. Marsh J. L. (2010). ERK activation by the polyphenols fisetin and resveratrol provides neuroprotection in multiple models of Huntington’s disease. Hum. Mol. Genet. 20, 261–270. 10.1093/hmg/ddq460 20952447 PMC3005900

[B171] Marcon G. Di Fede G. Giaccone G. Rossi G. Giovagnoli A. R. Maccagnano E. (2009). A novel Italian presenilin 2 gene mutation with prevalent behavioral phenotype. J. Alzheimers Dis. 16, 509–511. 10.3233/JAD-2009-0986 19276543

[B172] Marin O. Meggio F. Sarno S. Andretta M. Pinna L. A. (1994). Phosphorylation of synthetic fragments of inhibitor-2 of protein phosphatase-1 by casein kinase-1 and -2. Evidence that phosphorylated residues are not strictly required for efficient targeting by casein kinase-1. Eur. J. Biochem. 223, 647–653. 10.1111/j.1432-1033.1994.tb19037.x 8055935

[B173] Maroteaux L. Campanelli J. T. Scheller R. H. (1988). Synuclein: A neuron-specific protein localized to the nucleus and presynaptic nerve terminal. J. Neurosci. 8, 2804–2815. 10.1523/JNEUROSCI.08-08-02804.1988 3411354 PMC6569395

[B174] Martínez-González L. Rodríguez-Cueto C. Cabezudo D. Bartolomé F. Andrés-Benito P. Ferrer I. (2020). Motor neuron preservation and decrease of *in vivo* TDP-43 phosphorylation by protein CK-1δ kinase inhibitor treatment. Sci. Rep. 10, 4449. 10.1038/s41598-020-61265-y 32157143 PMC7064575

[B175] Mashhoon N. De Maggio A. J. Tereshko V. Bergmeier S. C. Egli M. Hoekstra M. F. (2000). Crystal structure of a conformation-selective casein kinase-1 inhibitor. J. Biol. Chem. 275, 20052–20060. 10.1074/jbcM001713200 10749871

[B176] Mbefo M. K. Fares M.-B. Paleologou K. Oueslati A. Yin G. Tenreiro S. (2015). Parkinson disease mutant E46K enhances α-synuclein phosphorylation in mammalian cell lines, in yeast, and *in vivo* . J. Biol. Chem. 290, 9412–9427. 10.1074/jbc.m114.610774 25657004 PMC4392248

[B177] Meggio F. Marin O. Pinna L. A. (1994). Substrate specificity of protein kinase CK2. Cell. Mol. Biol. Res. 40, 401–409. 7735314

[B178] Meggio F. Perich J. W. Marin O. Pinna L. A. (1992). The comparative efficiencies of the Ser(P)-Thr(P)- and Tyr(P)-residues as specificity determinants for casein kinase-1. Biochem. Biophys. Res. Commun. 182, 1460–1465. 10.1016/0006-291x(92)91898-z 1540189

[B179] Meggio F. Perich J. W. Reynolds E. C. Pinna L. A. (1991). A synthetic β-casein phosphopeptide and analogues as model substrates for casein kinase-1, a ubiquitous, phosphate directed protein kinase. FEBS Lett. 283, 303–306. 10.1016/0014-5793(91)80614-9 2044770

[B180] Meggio F. Pinna L. A. (2003). One-thousand-and-one substrates of protein kinase CK2? FASEB J. 17, 349–368. 10.1096/fj.02-0473rev 12631575

[B181] Meijer L. Borgne A. Mulner O. Chong J. P. Blow J. J. Inagaki N. (1997). Biochemical and cellular effects of roscovitine, a potent and selective inhibitor of the cyclin-dependent kinases cdc2, cdk2 and cdk5. Eur. J. Biochem. 243, 527–536. 10.1111/j.1432-1033.1997.t01-2-00527.x 9030781

[B182] Meijer L. Thunnissen A.-M. White A. Garnier M. Nikolic M. Tsai L.-H. (2000). Inhibition of cyclin-dependent kinases, GSK-3beta and CK1 by hymenialdisine, a marine sponge constituent. Chem. Biol. 7, 51–63. 10.1016/s1074-5521(00)00063-6 10662688

[B183] Meng Q.-J. Maywood E. S. Bechtold D. A. Lu W.-Q. Li J. Gibbs J. E. (2010). Entrainment of disrupted circadian behavior through inhibition of casein kinase 1 (CK1) enzymes. Proc. Natl. Acad. Sci. U. S. A. 107, 15240–15245. 10.1073/pnas.1005101107 20696890 PMC2930590

[B184] Menke R. A. Jbabdi S. Miller K. L. Matthews P. M. Zarei M. (2010). Connectivity-based segmentation of the substantia nigra in human and its implications in Parkinson's disease. Neuroimage 52, 1175–1180. 10.1016/j.neuroimage.2010.05.086 20677376

[B186] Mishra S. Kinoshita Ch. Axtman A. D. Young J. E. (2022). Evaluation of a selective chemical probe validates that CK2 mediates neuroinflammation in a human induced pluripotent stem cell-derived mircroglial model. Front. Mol. Neurosci. 15, 824956. 10.3389/fnmol.2022.824956 35774866 PMC9239073

[B187] Morales-Garcia J. A. Salado I. G. Sanz-San Cristobal M. Gil C. Pérez-Castillo A. Martínez A. (2017). Biological and pharmacological characterization of benzothiazole-based CK-1δ inhibitors in models of Parkinson’s disease. ACS Omega 2, 5215–5220. 10.1021/acsomega.7b00869 30023743 PMC6044573

[B188] Morooka S. Hoshina M. Kii I. Okabe T. Kojima H. Inoue N. (2015). Identification of a dual inhibitor of SRPK1 and CK2 that attenuates pathological angiogenesis of macular degeneration in mice. Mol. Pharmacol. 88, 316–325. 10.1124/mol.114.097345 25993998

[B189] Mueller T. Breuer P. Schmitt I. Walter J. Evert B. O. Wüllner U. (2009). CK2-dependent phosphorylation determines cellular localization and stability of ataxin-3. Hum. Mol. Genet. 18, 3334–3343. 10.1093/hmg/ddp274 19542537

[B190] Murphy M. P. LeVine H. (2010). Alzheimer’s disease and the amyloid-β peptide. J. Alzheimer’s Dis. 19, 311–323. 10.3233/jad-2010-1221 20061647 PMC2813509

[B191] Nakagawa T. Ohta K. (2019). Quercetin regulates the integrated stress response to improve memory. Int. J. Mol. Sci. 20, 2761. 10.3390/ijms20112761 PMC6600673 31195662

[B192] Nakamura T. Yamashita H. Takahashi T. Nakamura S. (2001). Activated Fyn phosphorylates alpha-synuclein at tyrosine residue 125. Biochem. Biophys. Res. Commun. 280, 1085–1092. 10.1006/bbrc.2000.4253 11162638

[B193] Nakielny S. Dreyfuss G. (1997). Nuclear export of proteins and RNAs. Curr. Opin. Cell Biol. 9, 420–429. 10.1016/s0955-0674(97)80016-6 9159083

[B194] Narhi L. Wood S. J. Steavenson S. Jiang Y. Wu G. M. Anafi D. (1999). Both familial Parkinson’s disease mutations accelerate alpha-synuclein aggregation. J. Biol. Chem. 274, 9843–9846. 10.1074/jbc.274.14.9843 10092675

[B195] Negro A. Brunati A. M. Donella-Deana A. Massimino M. L. Pinna L. A. (2002). Multiple phosphorylation of alpha-synuclein by protein tyrosine kinase Syk prevents eosin-induced aggregation. FASEB J. 16, 210–212. 10.1096/fj.01-0517fje 11744621

[B196] Neumann M. Bentmann E. Dormann D. Jawaid A. DeJesus-Hernandez M. Ansorge O. (2011). FET proteins TAF15 and EWS are selective markers that distinguish FTLD with FUS pathology from amyotrophic lateral sclerosis with FUS mutations. Brain 134, 2595–2609. 10.1093/brain/awr201 21856723 PMC3170539

[B197] Neumann M. Sampathu D. M. Kwong L. K. Truax A. C. Micsenyi M. C. Chou T. T. (2006). Ubiquitinated TDP-43 in frontotemporal lobar degeneration and amyotrophic lateral sclerosis. Science 314, 130–133. 10.1126/science.1134108 17023659

[B198] Nirmaladevi D. Venkataramana M. Chandranayaka S. Ramesha A. Jameel N. M. Srinivas C. (2014). Neuroprotective effects of bikaverin on H_2_O_2_-induced oxidative stress mediated neuronal damage in SH-SY5Y cell line. Cell. Mol. Neurobiol. 34, 973–985. 10.1007/s10571-014-0073-6 24848007 PMC11488908

[B199] Nishi H. Shaytan A. Panchenko A. R. (2014). Physicochemical mechanisms of protein regulation by phosphorylation. Front. Genet. 5, 270. 10.3389/fgene.2014.00270 25147561 PMC4124799

[B200] Nonaka T. Suzuki G. Tanaka Y. Kametani F. Hirai S. Okado H. (2016). Phosphorylation of TAR DNA-binding protein of 43 kDa (TDP-43) by truncated casein kinase 1 delta triggers mislocalization and accumulation of TDP-43. J. Biol. Chem. 291, 5473–5483. 10.1074/jbc.M115.695379 26769969 PMC4786690

[B201] Nozal V. Martinez A. (2019). Tau Tubulin Kinase 1 (TTBK1), a new player in the fight against neurodegenerative diseases. Eur. J. Med. Chem. 161, 39–47. 10.1016/j.ejmech.2018.10.030 30342424

[B202] Nozal V. Martínez-González L. Gomez-Almeria M. Gonzalo-Consuegra C. Santana P. Chaikuad A. (2022). TDP-43 modulation by tau-tubulin kinase 1 inhibitors: A new avenue for future amyotrophic lateral sclerosis therapy. J. Med. Chem. 65, 1585–1607. 10.1021/acs.jmedchem.1c01942 34978799

[B203] O’Brien R. J. Wong P. C. (2011). Amyloid precursor protein processing and Alzheimer’s disease. Annu. Rev. Neurosci. 34, 185–204. 10.1146/annurev-neuro-061010-113613 21456963 PMC3174086

[B204] Okochi M. Walter J. Koyama A. Nakajo S. Baba M. Iwatsubo T. (2000). Constitutive phosphorylation of the Parkinson’s disease associated alpha-synuclein. J. Biol. Chem. 275, 390–397. 10.1074/jbc.275.1.390 10617630

[B205] Oliveira J. Costa M. de Almeida M. S. C. da Cruz e Silva O. A. B. Henriques A. G. (2017). Protein phosphorylation is a key mechanism in Alzheimer’s disease. J. Alzheimer’s Dis. 58, 953–978. 10.3233/jad-170176 28527217

[B206] Oshima T. Niwa Y. Kuwata K. Srivastava A. Hyoda T. Tsuchiya Y. (2019). Cell-based screen identifies a new potent and highly selective CK2 inhibitor for modulation of circadian rhythms and cancer cell growth. Sci. Adv. 5, eaau9060. 10.1126/sciadv.aau9060 30746467 PMC6357737

[B207] Ou S. H. Wu F. Harrich D. Garcia-Martinez L. F. Gaynor R. B. (1995). Cloning and characterization of a novel cellular protein, TDP-43, that binds to human immunodeficiency virus type 1 TAR DNA sequence motifs. J. Virol. 69, 3584–3596. 10.1128/JVI.69.6.3584-3596.1995 7745706 PMC189073

[B208] Oueslati A. (2016). Implication of alpha-synuclein phosphorylation at S129 in synucleinopathies: What have we learned in the last decade? J. Park. Dis. 6, 39–51. 10.3233/JPD-160779 PMC4927808 27003784

[B209] Pagano M. A. Bain J. Kazimierczuk Z. Sarno S. Ruzzene M. Di Maira G. (2008). The selectivity of inhibitors of protein kinase CK2: An update. Biochem. J. 415, 353–365. 10.1042/bj20080309 18588507

[B210] Paleologou K. E. Schmid A. W. Rospigliosi C. C. Kim H.-Y. Lamberto G. R. Fredenburg R. A. (2008). Phosphorylation at ser-129 but not the phosphomimics S129E/D inhibits the fibrillation of α-synuclein. J. Biol. Chem. 283, 16895–16905. 10.1074/jbc.m800747200 18343814 PMC2423264

[B211] Palomo V. Nozal V. Rojas-Prats E. Gil C. Martinez A. (2020). Protein kinase inhibitors for amyotrophic lateral sclerosis therapy. Br. J. Pharmacol. 178, 1316–1335. 10.1111/bph.15221 32737989

[B212] Perez D. I. Gil C. Martinez A. (2011). Protein kinases CK1 and CK2 as new targets for neurodegenerative diseases. Med. Res. Rev. 31, 924–954. 10.1002/med.20207 20577972

[B213] Pierre F. Chua P. C. O’Brien S. E. Siddiqui-Jain A. Bourbon P. Haddach M. (2011). Pre-clinical characterization of CX-4945, a potent and selective small molecule inhibitor of CK2 for the treatment of cancer. Mol. Cell. Biochem. 356, 37–43. 10.1007/s11010-011-0956-5 21755459

[B214] Pinna L. A. (2002). Protein kinase CK2: A challenge to canons. J. Cell Sci. 115, 3873–3878. 10.1242/jcs.00074 12244125

[B215] Plisson F. Prasad P. Xiao X. Piggott A. M. Huang X. Khalil Z. (2014). Callyspongisines A–D: Bromopyrrole alkaloids from an Australian marine sponge, callyspongia sp. Org. Biomol. Chem. 12, 1579–1584. 10.1039/c4ob00091a 24458130

[B216] Posa D. Martinez-Gonzalez L. Bartolome F. Nagaraj S. Porras G. Martinez A. (2019). Recapitulation of pathological TDP-43 features in immortalized lymphocytes from sporadic ALS patients. Mol. Neurobiol. 56, 2424–2432. 10.1007/s12035-018-1249-8 30030753

[B217] Potjewyd F. M. Marquez A. B. Chaikuad A. Howel S. Dunn A. S. Beltran A. A. (2022). Modulation of tau tubulin kinases (TTBK1 and TTBK2) impacts ciliogenesis. bioRxiv 2022, 06490937. 10.1101/2022.05.06.490937 PMC10104807 37059819

[B218] Pulgar V. Marin O. Meggio F. Allende C. C. Allende J. E. Pinna L. A. (1999). Optimal sequences for non-phosphate-directed phosphorylation by protein kinase CK1 (casein kinase-1)--a re-evaluation. Eur. J. Biochem. 260, 520–526. 10.1046/j.1432-1327.1999.00195.x 10095790

[B219] Raux G. Gantier R. Martin C. Pothin Y. Brice A. Frebourg T. (2000). A novel presenilin 1 missense mutation (L153V) segregating with early-onset autosomal dominant Alzheimer’s disease. Hum. Mutat. 16, 95. 10.1002/1098-1004(200007)16:1<95:AID-HUMU28>3.0.CO;2-H 10874324

[B220] Regal L. Vanopdenbosch L. Tilkin P. Van den Bosch L. Thijs V. Sciot R. (2006). The G93C mutation in superoxide dismutase 1: Clinicopathologic phenotype and prognosis. Arch. Neurol. 63, 262–267. 10.1001/archneur.63.2.262 16476815

[B221] Reiner A. Albin R. L. Anderson K. D. D’Amato C. J. Penney J. B. Young A. B. (1988). Differential loss of striatal projection neurons in Huntington disease. Proc. Natl. Acad. Sci. U. S. A. 85, 5733–5737. 10.1073/pnas.85.15.5733 2456581 PMC281835

[B222] Rena G. Bain J. Elliott M. Cohen P. (2004). D4476, a cell-permeant inhibitor of CK1, suppresses the site-specific phosphorylation and nuclear exclusion of FOXO1a. EMBO Rep. 5, 60–65. 10.1038/sj.embor.7400048 14710188 PMC1298959

[B223] Renton A. E. Chiò A. Traynor B. J. (2013). State of play in amyotrophic lateral sclerosis genetics. Nat. Neurosci. 17, 17–23. 10.1038/nn.3584 24369373 PMC4544832

[B224] Rezai-Zadeh K. Shytle R. D. Bai Y. Tian J. Hou H. Mori T. (2009). Flavonoid-mediated presenilin-1 phosphorylation reduces Alzheimer's disease beta-amyloid production. J. Cell. Mol. Med. 13, 574–588. 10.1111/j.1582-4934.2008.00344.x 18410522 PMC2671567

[B225] Richter J. Bischof J. Zaja M. Kohlhof H. Othersen O. Vitt D. (2014). Difluoro-dioxolo-benzoimidazol-benzamides as potent inhibitors of CK1δ and ε with nanomolar inhibitory activity on cancer cell proliferation. J. Med. Chem. 57, 7933–7946. 10.1021/jm500600b 25191940

[B226] Ríos J.-L. Giner R. Marín M. Recio M. (2018). A pharmacological update of ellagic acid. Planta Med. 84, 1068–1093. 10.1055/a-0633-9492 29847844

[B227] Robberecht W. Philips T. (2013). The changing scene of amyotrophic lateral sclerosis. Nat. Rev. Neurosci. 14, 248–264. 10.1038/nrn3430 23463272

[B228] Rosenberger A. F. N. Morrema T. H. J. Gerritsen W. H. van Haastert E. S. Snkhchyan H. Hilhorst R. (2016). Increased occurrence of protein kinase CK2 in astrocytes in Alzheimer’s disease pathology. J. Neuroinflammation 13, 4. 10.1186/s12974-015-0470-x 26732432 PMC4702323

[B229] Rosenblatt A. (2007). Neuropsychiatry of Huntington’s disease. Dialogues Clin. Neurosci. 9, 191–197. 10.31887/DCNS.2007.9.2/arosenblatt 17726917 PMC3181855

[B231] Rubinsztein D. C. Leggo J. Coles R. Almqvist E. Biancalana V. Cassiman J. J. (1996). Phenotypic characterization of individuals with 30-40 CAG repeats in the Huntington disease (HD) gene reveals HD cases with 36 repeats and apparently normal elderly individuals with 36-39 repeats. Am. J. Hum. Genet. 59, 16–22. 8659522 PMC1915122

[B232] Rubio de la Torre E. Luzon-Toro B. Forte-Lago I. Minguez-Castellanos A. Ferrer I. Hilfiker S. (2009). Combined kinase inhibition modulates Parkin inactivation. Hum. Mol. Genet. 18, 809–823. 10.1093/hmg/ddn407 19050041 PMC2640208

[B233] Salado I. G. Redondo M. Bello M. L. Perez C. Liachko N. F. Kraemer B. C. (2014). Protein kinase CK-1 inhibitors as new potential drugs for amyotrophic lateral sclerosis. J. Med. Chem. 57, 2755–2772. 10.1021/jm500065f 24592867 PMC3969104

[B234] Salvi M. Sarno S. Cesaro L. Nakamura H. Pinna L. A. (2009). Extraordinary pleiotropy of protein kinase CK2 revealed by weblogo phosphoproteome analysis. Biochim. Biophys. Acta 1793, 847–859. 10.1016/j.bbamcr.2009.01.013 19339213

[B235] Sannerud R. Esselens C. Ejsmont P. Mattera R. Rochin L. Tharkeshwar A. K. (2016). Restricted location of PSEN2/γ-secretase determines substrate specificity and generates an intracellular Aβ pool. Cell 166, 193–208. 10.1016/j.cell.2016.05.020 27293189 PMC7439524

[B236] Sarno S. Reddy H. Meggio F. Ruzzene M. Davies S. P. Donella-Deana A. (2001). Selectivity of 4, 5, 6, 7-tetrabromobenzotriazole, an ATP site-directed inhibitor of protein kinase CK2 (“casein kinase-2”). FEBS Lett. 496, 44–48. 10.1016/s0014-5793(01)02404-8 11343704

[B237] Sato N. Hori O. Yamaguchi A. Lambert J. C. Chartier-Harlin M. C. Robinson P. A. (1999). A novel presenilin-2 splice variant in human Alzheimer’s disease brain tissue. J. Neurochem. 72, 2498–2505. 10.1046/j.1471-4159.1999.0722498.x 10349860

[B238] Sato S. Cerny R. L. Buescher J. L. Ikezu T. (2006). Tau-tubulin kinase 1 (TTBK1), a neuron-specific tau kinase candidate, is involved in tau phosphorylation and aggregation. J. Neurochem. 98, 1573–1584. 10.1111/j.1471-4159.2006.04059.x 16923168

[B239] Sato S. Xu J. Okuyama S. Martinez L. B. Walsh S. M. Jacobsen M. T. (2008). Spatial learning impairment, enhanced CDK5/p35 activity, and downregulation of NMDA receptor expression in transgenic mice expressing tau-tubulin kinase 1. J. Neurosci. 28, 14511–14521. 10.1523/jneurosci.3417-08.2008 19118186 PMC6671237

[B240] Schwab C. DeMaggio A. J. Ghoshal N. Binder L. I. Kuret J. McGeer P. L. (2000). Casein kinase 1 delta is associated with pathological accumulation of tau in several neurodegenerative diseases. Neurobiol. Aging 21, 503–510. 10.1016/s0197-4580(00)00110-x 10924763

[B241] Schwartz P. A. Murray B. W. (2011). Protein kinase biochemistry and drug discovery. Bioorg. Chem. 39, 192–210. 10.1016/j.bioorg.2011.07.004 21872901

[B242] Schwind L. Nalbach L. Zimmer A. D. Kostelnik K. B. Menegatti J. Grässer F. (2017). Quinalizarin inhibits adipogenesis through down-regulation of transcription factors and microRNA modulation. Biochim. Biophys. Acta. Gen. Subj. 1861, 3272–3281. 10.1016/j.bbagen.2017.09.018 28964816

[B243] Selvakumar K. Bavithra S. Suganthi M. Benson C. S. Elumalai P. Arunkumar R. (2012). Protective role of quercetin on PCBs-induced oxidative stress and apoptosis in hippocampus of adult rats. Neurochem. Res. 37, 708–721. 10.1007/s11064-011-0661-5 22127757

[B244] Shao G. Chen S. Sun Y. Xu H. Ge F. (2021). Chrysoeriol promotes functional neurological recovery in a rat model of cerebral ischemia. Phcog. Mag. 17, 802–810. 10.4103/pm.pm_329_21

[B245] Sharma V. Mishra M. Ghosh S. Tewari R. Basu A. Seth P. (2007). Modulation of interleukin-1beta mediated inflammatory response in human astrocytes by flavonoids: Implications in neuroprotection. Brain Res. Bull. 73, 55–63. 10.1016/j.brainresbull.2007.01.016 17499637

[B246] Shimura H. Hattori N. Kubo S. Mizuno Y. Asakawa S. Minoshima S. (2000). Familial Parkinson disease gene product, parkin, is a ubiquitin-protein ligase. Nat. Genet. 25, 302–305. 10.1038/77060 10888878

[B247] Siddiqui-Jain A. Drygin D. Streiner N. Chua P. Pierre F. O'Brien S. E. (2010). CX-4945, an orally bioavailable selective inhibitor of protein kinase CK2, inhibits prosurvival and angiogenic signaling and exhibits antitumor efficacy. Cancer Res. 70, 10288–10298. 10.1158/0008-5472.CAN-10-1893 21159648

[B248] Smith M. J. Sharples R. A. Evin G. McLean C. A. Dean B. Pavey G. (2004). Expression of truncated presenilin 2 splice variant in Alzheimer’s disease, bipolar disorder, and schizophrenia brain cortex. Brain Res. Mol. Brain Res. 127, 128–135. 10.1016/j.molbrainres.2004.05.019 15306129

[B249] Spillantini M. G. Schmidt M. L. Lee V. M. Trojanowski J. Q. Jakes R. Goedert M. (1997). Alpha-synuclein in Lewy bodies. Nature 388, 839–840. 10.1038/42166 9278044

[B250] Sreedharan J. Blair I. P. Tripathi V. B. Hu X. Vance C. Rogelj B. (2008). TDP-43 mutations in familial and sporadic amyotrophic lateral sclerosis. Science 319, 1668–1672. 10.1126/science.1154584 18309045 PMC7116650

[B251] Sriram S. R. Li X. Ko H. S. Chung K. K. Wong E. Lim K. L. (2005). Familial-associated mutations differentially disrupt the solubility, localization, binding and ubiquitination properties of parkin. Hum. Mol. Genet. 14, 2571–2586. 10.1093/hmg/ddi292 16049031

[B252] Su C.-F. Jiang L. Zhang X. W. Iyaswamy A. Li M. (2021). Resveratrol in rodent models of Parkinson’s disease: A systematic review of experimental studies. Front. Pharmacol. 12, 644219. 10.3389/fphar.2021.644219 33967780 PMC8100515

[B253] Su Z. Song J. Wang Z. Zhou L. Xia Y. Yu S. (2018). Tumor promoter TPA activates Wnt/β-catenin signaling in a casein kinase 1-dependent manner. Proc. Natl. Acad. Sci. U. S. A. 115, E7522–E7531. 10.1073/pnas.1802422115 30038030 PMC6094128

[B254] Sundaram S. Nagaraj S. Mahoney H. Portugues A. Li W. Millsaps K. (2019). Inhibition of casein kinase 1δ/ε improves cognitive-affective behavior and reduces amyloid load in the APP-PS1 mouse model of Alzheimer’s disease. Sci. Rep. 9, 13743. 10.1038/s41598-019-50197-x 31551449 PMC6760153

[B255] Szyszka R. Grankowski N. Felczak K. Shugar D. (1995). Halogenated benzimidazoles and benzotriazoles as selective inhibitors of protein kinases CK-I and CK-ii from Saccharomyces cerevisiae and other sources. Biochem. Biophys. Res. Commun. 208, 418–424. 10.1006/bbrc.1995.1354 7887958

[B256] Tagliabracci V. S. Wiley S. E. Guo X. Kinch L. N. Durrant E. Wen J. (2015). A single kinase generates the majority of the secreted phosphoproteome. Cell 161, 1619–1632. 10.1016/j.cell.2015.05.028 26091039 PMC4963185

[B257] Takahashi M. Tomizawa K. Sato K. Ohtake A. Omori A. (1995). A novel tau-tubulin kinase from bovine brain. FEBS Lett. 372, 59–64. 10.1016/0014-5793(95)00955-9 7556643

[B258] Takano A. Hoe H.-S. Isojima Y. Nagai K. (2004). Analysis of the expression, localization and activity of rat casein kinase 1epsilon-3. Neuroreport 15, 1461–1464. 10.1097/01.wnr.0000133297.77278.81 15194874

[B259] Takashima A. Noguchi K. Sato K. Hoshomoto T. Imahori K. (1993). Tau protein kinase I is essential for amyloid beta-protein-induced neurotoxicity. Proc. Natl. Acad. Sci. U. S. A. 90, 7789–7793. 10.1073/pnas.90.16.7789 8356085 PMC47228

[B260] Tanimukai H. Grundke-Iqbal I. Iqbal K. (2005). Up-regulation of inhibitors of protein phosphatase-2A in Alzheimer’s disease. Am. J. Pathol. 66, 1761–1771. 10.1016/S0002-9440(10)62486-8 PMC1602412 15920161

[B261] Tarrant M. K. Cole P. A. (2009). The chemical biology of protein phosphorylation. Annu. Rev. Biochem. 78, 797–825. 10.1146/annurev.biochem.78.070907.103047 19489734 PMC3074175

[B262] Taylor L. M. McMillan P. J. Kraemer B. C. Liachko N. F. (2019). Tau tubulin kinases in proteinopathy. FEBS J. 286, 2434–2446. 10.1111/febs.14866 31034749 PMC6936727

[B263] Taylor L. M. McMillan P. J. Liachko N. F. Strovas T. J. Ghetti B. Bird T. D. (2018). Pathological phosphorylation of tau and TDP-43 by TTBK1 and TTBK2 drives neurodegeneration. Mol. Neurodegener. 13, 7. 10.1186/s13024-018-0237-9 29409526 PMC5802059

[B264] Taylor S. S. Kornev A. P. (2011). Protein kinases: Evolution of dynamic regulatory proteins. Trends biochem. Sci. 36, 65–77. 10.1016/j.tibs.2010.09.006 20971646 PMC3084033

[B265] Taylor S. S. Radzio-Andzelm E. Hunter T. (1995). How do protein kinases discriminate between serine/threonine and tyrosine? Structural insights from the insulin receptor protein-tyrosine kinase. FASEB J. 9, 1255–1266. 10.1096/fasebj.9.13.7557015 7557015

[B266] Tian Y. Wang Y. Jablonski A. M. Hu Y. Sugam J. A. Koglin M. (2021). Tau-tubulin kinase 1 phosphorylates TDP-43 at disease-relevant sites and exacerbates TDP-43 pathology. Neurobiol. Dis. 161, 105548. 10.1016/j.nbd.2021.105548 34752923

[B267] Tiwari S. Atluri V. Kaushik A. Yndar T. A. Nair M. (2019). Alzheimer’s disease: Pathogenesis, diagnostics, and therapeutics. Int. J. Nanomedicine 14, 5541–5554. 10.2147/IJN.S200490 31410002 PMC6650620

[B268] Tomizawa K. Omori A. Ohtake A. Sato K. Takahashi M. (2001). Tau-tubulin kinase phosphorylates tau at Ser-208 and Ser-210, sites found in paired helical filament-tau. FEBS Lett. 492, 221–227. 10.1016/s0014-5793(01)02256-6 11257498

[B269] Turner R. S. Thomas R. G. Craft S. van Dyck C. H. Mintzer J. Reynolds B. A. (2015). A randomized, double-blind, placebo-controlled trial of resveratrol for Alzheimer disease. Neurology 85, 1383–1391. 10.1212/wnl.0000000000002035 26362286 PMC4626244

[B270] Ueda K. Fukushima H. Masliah E. Xia Y. Iwai A. Yoshimoto M. (1993). Molecular cloning of cDNA encoding an unrecognized component of amyloid in Alzheimer disease. Proc. Natl. Acad. Sci. U. S. A. 90, 11282–11286. 10.1073/pnas.90.23.11282 8248242 PMC47966

[B271] Ullah A. Munir S. Badshah S. L. Khan N. Ghani L. Poulson B. G. (2020). Important flavonoids and their role as a therapeutic agent. Molecules 25, 5243. 10.3390/molecules25225243 PMC7697716 33187049

[B272] Uversky V. N. (2007). Neuropathology, biochemistry, and biophysics of alpha-synuclein aggregation. J. Neurochem. 103, 17–37. 10.1111/j.1471-4159.2007.04764.x 17623039

[B273] Varela L. Garcia-Rendueles M. E. R. (2022). Oncogenic pathways in neurodegenerative diseases. Int. J. Mol. Sci. 23, 3223. 10.3390/ijms23063223 35328644 PMC8952192

[B274] Vargas M. R. Pehar M. Cassina P. Beckman J. S. Barbeito L. (2006). Increased glutathione biosynthesis by Nrf2 activation in astrocytes prevents p75NTR-dependent motor neuron apoptosis. J. Neurochem. 97, 687–696. 10.1111/j.1471-4159.2006.03742.x 16524372

[B275] Vassar R. Bennett B. D. Babu-Kahn S. Kahn S. Mendiaz E. A. Denis P. (1999). Beta-secretase cleavage of Alzheimer's amyloid precursor protein by the transmembrane aspartic protease BACE. Science 286, 735–741. 10.1126/science.286.5440.735 10531052

[B276] Vazquez-Higuera J. L. Martinez-Garcia A. Sanchez-Juan P. Rodriguez-Rodriguez E. Mateo I. Pozueta A. (2011). Genetic variations in tau-tubulin kinase-1 are linked to Alzheimer’s disease in a Spanish case-control cohort. Neurobiol. Aging 32, e5–9. 10.1016/j.neurobiolaging.2009.12.021 20096481

[B277] Venerando A. Ruzzene M. Pinna L. A. (2014). Casein kinase: The triple meaning of a misnomer. Biochem. J. 460, 141–156. 10.1042/bj20140178 24825444

[B278] Vera J. Estanyol J. M. Canela N. Llorens F. Agell N. Itarte E. (2007). Proteomic analysis of SET-binding proteins. Proteomics 7, 578–587. 10.1002/pmic.200600458 17309103

[B279] Versluys L. Pereira P. E. Schuermans N. De Paepe B. De Bleecker J. L. Bogaert E. (2022). Expanding the TDP-43 proteinopathy pathway from neurons to muscle: Physiological and pathophysiological functions. Front. Neurosci. 16, 815765. 10.3389/fnins.2022.815765 35185458 PMC8851062

[B280] Waddy C. T. Mackinlay A. G. (1971). Protein kinase activity from lactating bovine mammary gland. Biochim. Biophys. Acta 250, 491–500. 10.1016/0005-2744(71)90249-x 4332212

[B281] Wager T. T. Chandrasekaran R. Y. Bradley J. Rubitski D. Berke H. Mente S. (2014). Casein kinase 1δ/ε inhibitor PF-5006739 attenuates opioid drug-seeking behavior. ACS Chem. Neurosci. 5, 1253–1265. 10.1021/cn500201x 25299732

[B282] Wakabayashi K. Matsumoto K. Takayama K. Yoshimoto M. Takahashi H. (1997). NACP, a presynaptic protein, immunoreactivity in Lewy bodies in Parkinson’s disease. Neurosci. Lett. 239, 45–48. 10.1016/s0304-3940(97)00891-4 9547168

[B283] Walter J. Capell A. Grünberg J. Pesold B. Schindzielorz A. Prior R. (1996). The Alzheimer's disease-associated presenilins are differentially phosphorylated proteins located predominantly within the endoplasmic reticulum. Mol. Med. 2, 673–691. 10.1007/BF03401652 8972483 PMC2230134

[B284] Walter J. Fluhrer R. Hartung B. Willem M. Kaether C. Capell A. (2001). Phosphorylation regulates intracellular trafficking of β-secretase. J. Biol. Chem. 276, 14634–14641. 10.1074/jbc.m011116200 11278841

[B285] Walter J. Schindzielorz A. Hartung B. Haass C. (2000). Phosphorylation of the beta-amyloid precursor protein at the cell surface by ectocasein kinases 1 and 2. J. Biol. Chem. 275, 23523–23529. 10.1074/jbc.M002850200 10806211

[B286] Walter J. (2000). The phosphorylation of presenilin proteins. Methods Mol. Med. 32, 317–331. 10.1385/1-59259-195-7:317 21318529

[B287] Walton K. M. Fisher K. Rubitski D. Marconi M. Meng Q.-J. Sladek M. (2009). Selective inhibition of casein kinase 1 minimally alters circadian clock period. J. Pharmacol. Exp. Ther. 330, 430–439. 10.1124/jpet.109.151415 19458106

[B288] Wan Y. Hur W. Cho C. Y. Liu Y. Adrian F. J. Lozach O. (2004). Synthesis and target identification of hymenialdisine analogs. Chem. Biol. 11, 247–259. 10.1016/j.chembiol.2004.01.015 15123286

[B289] Wang B. Lu Z. Polya G. (1998). Inhibition of eukaryote serine/threonine-specific protein kinases by piceatannol. Planta Med. 64, 195–199. 10.1055/s-2006-957407 9581512

[B290] Wang Q. Dong X. Zhang R. Zhao Ch. (2021). Flavonoids with potential anti-amyloidogenic effects as therapeutic drugs for treating alzheimer's disease. J. Alzheimers Dis. 84, 505–533. 10.3233/JAD-210735 34569961

[B291] Wang S.-H. Liang C.-H. Liang F.-P. Ding H.-Y. Lin S.-P. Huang G.-J. (2016). The inhibitory mechanisms study of 5, 6, 4′-trihydroxy-7, 3′-dimethoxyflavone against the LPS-induced macrophage inflammatory responses through the antioxidant ability. Molecules 21, 136. 10.3390/molecules21020136 26805809 PMC6274540

[B292] Waxman E. A. Giasson B. I. (2008). Specificity and regulation of casein kinase/mediated phosphorylation of α-synuclein. J. Neuropathol. Exp. Neurol. 67, 402–416. 10.1097/NEN.0b013e31816fc995 18451726 PMC2930078

[B293] Wei Y. Zhu G. Zheng C. Li J. Sheng S. Li D. (2020). Ellagic acid protects dopamine neurons from rotenone-induced neurotoxicity via activation of Nrf2 signalling. J. Cell. Mol. Med. 24, 9446–9456. 10.1111/jcmm.15616 32657027 PMC7417702

[B294] Weingarten M. D. Lockwood A. H. Hwo S. Y. Kirschner M. W. (1975). A protein factor essential for microtubule assembly. Proc. Natl. Acad. Sci. U. S. A. 72, 1858–1862. 10.1073/pnas.72.5.1858 1057175 PMC432646

[B295] Wells C. I. Drewry D. H. Pickett J. E. Tjaden A. Krämer A. Müller S. (2021). Development of a potent and selective chemical probe for the pleiotropic kinase CK2. Cell Chem. Biol. 28, 546–558. e10. 10.1016/j.chembiol.2020.12.013 33484635 PMC8864761

[B296] Wirkner U. Voss H. Lichter P. Ansorge W. Pyerin W. (1994). The human gene (CSNK2A1) coding for the casein kinase II subunit alpha is located on chromosome 20 and contains tandemly arranged Alu repeats. Genomics 19, 257–265. 10.1006/geno.1994.1056 8188256

[B297] Xia J. Lee D. H. Taylor J. Vandelft M. Truant R. (2003). Huntingtin contains a highly conserved nuclear export signal. Hum. Mol. Genet. 12, 1393–1403. 10.1093/hmg/ddg156 12783847

[B298] Xu J. Sato S. Okuyama S. Swan R. J. Jacobsen M. T. Strunk E. (2010). Tau-tubulin kinase 1 enhances prefibrillar tau aggregation and motor neuron degeneration in P301L FTDP-17 tau-mutant mice. FASEB J. 24, 2904–2915. 10.1096/fj.09-150144 20354135

[B299] Xu P. Ianes C. Gärtner F. Liu C. Burster T. Bakulev V. (2019). Structure, regulation, and (patho-)physiological functions of the stress-induced protein kinase CK1 delta (CSNK1D). Gene 715, 144005. 10.1016/j.gene.2019.144005 31376410 PMC7939460

[B300] Xu Y. Deng Y. Qing H. (2015). The phosphorylation of α-synuclein: Development and implication for the mechanism and therapy of the Parkinson's disease. J. Neurochem. 135, 4–18. 10.1111/jnc.13234 26134497

[B301] Xue Y. Wan P. T. Hillertz P. Schweikart F. Zhao Y. Wissler L. (2013). X-ray structural analysis of tau-tubulin kinase 1 and its interactions with small molecular inhibitors. ChemMedChem 8, 1846–1854. 10.1002/cmdc.201300274 24039150

[B302] Yadikar H. Torres I. Aiello G. Kurup M. Yang Z. Lin F. (2020). Screening of tau protein kinase inhibitors in a tauopathy-relevant cell-based model of tau hyperphosphorylation and oligomerization. PLOS ONE 15, e0224952. 10.1371/journal.pone.0224952 32692785 PMC7373298

[B303] Yamamoto A. Friedlein A. Imai Y. Takahashi R. Kahle P. J. Haass Ch. (2005). Parkin phosphorylation and modulation of its E3 ubiquitin ligase activity. J. Biol. Chem. 280, 3390–3399. 10.1074/jbc.M407724200 15557340

[B304] Yang A. J. T. Bagit A. MacPherson R. E. K. (2021). Resveratrol, metabolic dysregulation, and Alzheimer’s disease: Considerations for neurogenerative disease. Int. J. Mol. Sci. 22, 4628. 10.3390/ijms22094628 33924876 PMC8125227

[B305] Yang Y. Xu T. Zhang Y. Qin X. (2017). Molecular basis for the regulation of the circadian clock kinases CK1δ and CK1ε. Cell. Signal. 31, 58–65. 10.1016/j.cellsig.2016.12.010 28057520

[B306] Yang-Feng T. L. Naiman T. Kopatz I. Eli D. Dafni N. Canaani D. (1994). Assignment of the human casein kinase II alpha' subunit gene (CSNK2A1) to chromosome 16p13.2-p13.3. Genomics 19, 173. 10.1006/geno.1994.1032 8188223

[B307] Yasojima K. Schwab C. McGeer E. G. McGeer P. L. (2000). Human neurons generate C-reactive protein and amyloid P: Upregulation in Alzheimer’s disease. Brain Res. 887, 80–89. 10.1016/s0006-8993(00)02970-x 11134592

[B308] Yde C. W. Frogne T. Lykkesfeldt A. E. Fitchtner I. Issinger O.-G. Stenvang J. (2007). Induction of cell death in antiestrogen resistant human breast cancer cells by the protein kinase CK2 inhibitor DMAT. Cancer Lett. 256, 229–237. 10.1016/j.canlet.2007.06.010 17629615

[B309] Yonetani M. Nonaka T. Masuda M. Inukai Y. Oikawa T. Hisanaga S. (2009). Conversion of wildtype alpha-synuclein into mutant-type fibrils and its propagation in the presence of A30P mutant. J. Biol. Chem. 284, 7940–7950. 10.1074/jbc.M807482200 19164293 PMC2658087

[B310] Yoshida H. Ihara Y. (1993). Tau in paired helical filaments is functionally distinct from fetal tau: Assembly incompetence of paired helical filament-tau. J. Neurochem. 61, 1183–1186. 10.1111/j.1471-4159.1993.tb03642.x 8360683

[B311] Yu G. Yan T. Feng Y. Liu X. Xia Y. Luo H. (2013). Ser9 phosphorylation causes cytoplasmic detention of I_2_ ^PP2A^/SET in Alzheimer disease. Neurobiol. Aging 34, 1748–1758. 10.1016/j.neurobiolaging.2012.12.025 23374587

[B312] Yu N.-N. Yu J.-T. Xiao J.-T. Zhang H.-W. Lu R.-C. Jiang H. (2011). Tau-tubulin kinase-1 gene variants are associated with Alzheimer’s disease in Han Chinese. Neurosci. Lett. 491, 83–86. 10.1016/j.neulet.2011.01.011 21219968

[B313] Yu P. Wang L. Tang F. Zeng L. Zhou L. Song X. (2016). Resveratrol pretreatment decreases ischemic injury and improves neurological function via sonic hedgehog signaling after stroke in rats. Mol. Neurobiol. 54, 212–226. 10.1007/s12035-015-9639-7 26738852

[B314] Zaplatic E. Bule M. Shah S. Z. A. Uddin M. S. Niaz K. (2019). Molecular mechanisms underlying protective role of quercetin in attenuating Alzheimer’s disease. Life Sci. 224, 109–119. 10.1016/j.lfs.2019.03.055 30914316

[B315] Zhang H. Khalil Z. Conte M. M. Plisson F. Capon R. J. (2012). A search for kinase inhibitors and antibacterial agents: Bromopyrrolo-2-aminoimidazoles from a deep-water Great Australian Bight sponge *Axinella* sp. Tetrahedron Lett. 53, 3784–3787. 10.1016/j.tetlet.2012.05.051

[B316] Zhang J. Gross S. D. Schroeder M. D. Anderson R. A. (1996). Casein kinase I α and αL: Alternative splicing-generated kinases exhibit different catalytic properties. Biochemistry 35, 16319–16327. 10.1021/bi9614444 8973207

[B317] Zhang N. Gordon S. L. Fritsch M. J. Esoof N. Campbell D. G. Gourlay R. (2015). Phosphorylation of synaptic vesicle protein 2A at Thr84 by casein kinase 1 family kinases controls the specific retrieval of synaptotagmin-1. J. Neurosci. 35, 2492–2507. 10.1523/JNEUROSCI.4248-14.2015 25673844 PMC4323530

[B318] Zhang Q. Xia Y. Wang Y. Shentu Y. Zeng K. Mahaman Y. A. R. (2018). CK2 phosphorylating I_2_ ^PP2A^/SET mediates tau pathology and cognitive impairment. Front. Mol. Neurosci. 11, 146. 10.3389/fnmol.2018.00146 29760653 PMC5936753

[B319] Zhang Y.-J. Xu Y.-F. Cook C. Gendron T. F. Roettges P. Link C. D. (2009). Aberrant cleavage of TDP-43 enhances aggregation and cellular toxicity. Proc. Natl. Acad. Sci. U. S. A. 106, 7607–7612. 10.1073/pnas.0900688106 19383787 PMC2671323

[B320] Zhao H. Mei X. Yang D. Tu G. (2021). Resveratrol inhibits inflammation after spinal cord injury via SIRT-1/NF-κB signaling pathway. Neurosci. Lett. 762, 136151. 10.1016/j.neulet.2021.136151 34352338

[B322] Zhao Z. Wang L. Volk A. G. Birch N. W. Stoltz K. L. Bartom E. T. (2018). Regulation of MLL/COMPASS stability through its proteolytic cleavage by taspase1 as a possible approach for clinical therapy of leukemia. Genes Dev. 33, 61–74. 10.1101/gad.319830.118 30573454 PMC6317322

[B323] Zheng H. Koo E. H. (2006). The amyloid precursor protein: Beyond amyloid. Mol. Neurodegener. 1, 5. 10.1186/1750-1326-1-5 16930452 PMC1538601

[B324] Zheng J. C. Chen S. (2022). Translational Neurodegeneration in the era of fast growing international brain research. Transl. Neurodegener. 11, 1. 10.1186/s40035-021-00276-9 34974845 PMC8720459

[B325] Zhou Y. Li K. Zhang S. Li Q. Li Z. Zhou F. (2015). Quinalizarin, a specific CK2 inhibitor, reduces cell viability and suppresses migration and accelerates apoptosis in different human lung cancer cell lines. Indian J. Cancer 52, e119–e124. 10.4103/0019-509X.172508 26728669

